# Dealcoholized wine: Techniques, sensory impacts, stability, and perspectives of a growing industry

**DOI:** 10.1111/1541-4337.70171

**Published:** 2025-04-17

**Authors:** Wasim Akhtar, Adriana Teresa Ceci, Edoardo Longo, Marco Adolfo Marconi, Francesco Lonardi, Emanuele Boselli

**Affiliations:** ^1^ Oenolab, NOI Techpark, Faculty of Agricultural, Environmental and Food Sciences Free University of Bozen‐Bolzano Bozen‐Bolzano Italy; ^2^ R&D Department, Ju.Cla.S s.r.l Settimo di Pescantina Verona Verona Italy; ^3^ International Competence Center on Food Fermentations, Faculty of Agricultural, Environmental and Food Sciences Free University of Bozen‐Bolzano Bozen‐Bolzano Italy

**Keywords:** dealcoholization, dealcoholized wine, sensory impacts, wine market

## Abstract

The category of dealcoholized wine is receiving mounting interest within the wine industry related to the ability to retain sensory characteristics similar to regular wine while reducing or completely removing the alcohol level. This option has led health‐conscious consumers to seek a lower alcohol alternative without compromising the authentic wine experience. This review provides a comprehensive overview of the various dealcoholization techniques that are being used in the production of dealcoholized and partial dealcoholized wine, specifically examining reverse osmosis, osmotic distillation, vacuum distillation, spinning cone column, pervaporation, and diafiltration along with the effects of these methods on chemical and sensory characteristics of wine, involving flavor, aroma, mouthfeel, and finish. Various aspects of the impact of dealcoholization on wine stability were explored, including chemical, microbial, oxidative, and color stability. Furthermore, the market analysis of dealcoholized wine products including present and future growth in different regions is reported. Understanding these factors is of utmost importance for dealcoholized wine's growing advancement and market success, as it endeavors to accommodate various customer demands and preferences in a swiftly changing beverage environment.

**Abbreviations**
CFIA: Canadian Food Inspection Agency
FDA: Food and Drug Administration
FO: forward osmosis
FSANZ: Food Standards Australia New Zealand
EC: European Commission
EU: European Union
GC‐MS: gas chromatography‐mass spectrometry
IWSR: International Wine and Spirit Record
OD: osmotic distillation
OIV: International Organization of Vine and Wine
RO: reverse osmosis
RO‐EP: reverse osmosis‐evaporation pertraction
SCC: spinning cone column
USA: United States of America
VD: vacuum distillation

## 1 INTRODUCTION

In recent years, there has been a growing trend toward the dealcoholization of traditionally alcoholic wines to produce wines with a lower alcoholic strength and alcohol‐free wine. This trend is due to social pressures associated with road safety, the impact of alcohol consumption on the healthcare system due to the associated medical costs of chronic diseases, and a burgeoning non‐alcoholic wine sector which is estimated to be worth more than US$10 billion worldwide as reported by Ma et al. (2022) from market research report 2020. Moreover, growing consumer interest in lower alcohol wines is driven by social trends promoting reduced alcohol intake due to health concerns (Deroover et al., 2021; Nicholls, 2022; Rai et al., 2025). From this perspective, wine drinkers can still benefit from bioactive compounds such as vitamins, minerals, antioxidants, and anticancer agents while consuming wines with reduced alcohol content (Mangindaan et al., 2018).

Winemakers are employing alcohol management techniques to address rising ethanol levels in wine observed over the past three decades. High ethanol concentrations often enhance the perception of warmth on the palate, a trait generally seen as undesirable and unfavorable to wine quality. Globally, winemakers are increasingly adopting strategies to regulate or adjust ethanol content, and the viticulture management practices employed in vineyards are geared toward minimizing grape sugar levels (Olego et al., 2016; Pham et al., 2020), while the winery employs strategies that are aimed at limiting ethanol production during the fermentation process (Dequin et al., 2017; Rolle et al., 2018) or removing ethanol at a post‐fermentation step (Gil et al., 2013).

EU Regulation, 2021/2117, which amended the Common Market Organization (CMO), allows the production of still, sparkling, and semi‐sparkling wines with an alcohol content below 8.5% vol, including dealcoholized and partially dealcoholized wine. These wines originate from fermented grape musts, undergoing a winemaking process before alcohol removal (EP & CEU, 2021). The use of denominations “dealcoholized wine” and “partially dealcoholized wine” depends on the legislation of each state. Since 2012, the OIV (International Organization of Vine and Wine) has adopted two definitions: “Beverages obtained by wine dealcoholization” for those beverages with an alcohol content lower than 0.5% vol and “Beverages obtained by partial wine dealcoholization” for those in the range of 0.5%–8.5% vol (OIV‐ECO 432–2012, OIV‐ECO 433–2012 Resolutions, respectively) (Liguori et al., 2019; Ruf, 2013). A detailed overview of regulations is provided in Table 1 from world major wine‐producing and wine‐consuming regions.

**TABLE 1:** Regulations on permitted alcohol levels for dealcoholized and partially dealcoholized wines across major wine‐producing and wine‐consuming regions including the United States, Europe, Canada, the United Kingdom, South Africa, and Australia/New Zealand.

Countries/legislative bodies	Dealcoholized (alcohol level)	Partially dealcoholized/low alcoholic (alcohol level)	Non‐alcoholic/alcohol‐free/non‐intoxicating	References
USA (FDA)	≤0.5% (v/v)	<7 to >0.5% (v/v) (unspecific)	Negligible alcohol level	(Compliance Policy Guide [CGP], 2005)
European Union (EC)	≤0.5% (v/v)	>0.5% (v/v) to less than the level before dealcoholizing	ND	(EP & CEU, 2023)
Canada (CFIA)	<1.1% (v/v)	>1.1% (v/v)	<0.05% (v/v)	(Government of Canada, 2024)
UK	<0.5% (v/v)	≤1.2% (v/v)	<0.05% (v/v)	(UK, 2023)
South America	ND	ND	ND	–
South Africa	≤0.5% (v/v)	>0.5 to 4.5% (v/v)	<0.05% (v/v)	(South African Government, 1989)
Australia/New Zealand (FSANZ)	ND	<1.15% (v/v)	<0.5% (v/v) (non‐intoxicating)	(ANZFA, 2002)

*Note*: The data highlight variations in regulatory definitions, with specific thresholds for each category, reflecting differences in legislative approaches to alcohol‐free and reduced‐alcohol wine classifications.

Abbreviation: ND, not defined.

The management of alcohol consumption can pose a considerable challenge. In a recent review article, Kumar et al. (2024) reported that dealcoholization significantly affects a wine's volatile profile and sensory characteristics, varying based on the technique used, degree of alcohol reduction, and the physical and chemical properties of volatile compounds. While some volatiles and phenolics are retained at lower ethanol removal levels, dealcoholized wines generally show diminished aroma, flavor balance, and overall sensory quality. This is largely due to the loss of key esters, such as ethyl octanoate, ethyl acetate, and isoamyl acetate, which are essential for enhancing aroma perception (Longo et al., 2017). The process has been noted to heighten the perception of bitterness, diminish sourness, potentially modify the perception of sweetness, and decrease astringency, except when present alongside elevated levels of tannins. Furthermore, alcohol also masks esters in wines, reducing fruitiness (King et al., 2013). The potential decline in consumer acceptance and purchasing behavior due to volatile compound losses highlights the need for innovative approaches to reduce alcohol in wine while maintaining its sensory attributes (Longo et al., 2017).

**FIGURE 1:**
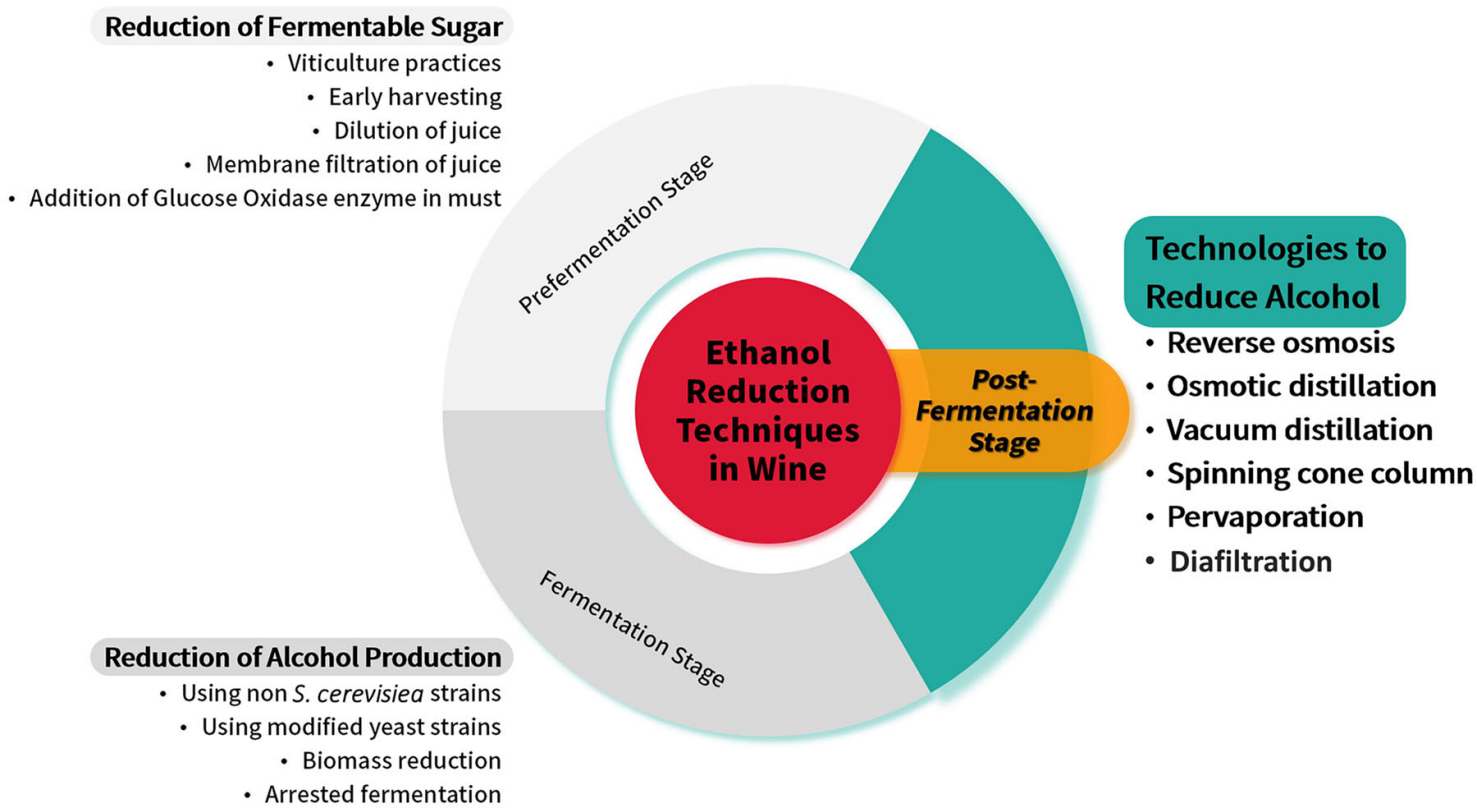
Illustration of the ethanol reduction techniques in wine, with a focus on post‐fermentation techniques, which involve methods applied after fermentation to selectively remove alcohol while maintaining wine quality.

The production of low‐alcohol beverages presents significant challenges despite increasing global demand. Manufacturers can use low‐sugar grapes, arrest fermentation with specialized yeast, or opt a non‐fermented approach blending with aroma compounds. However, these approaches negatively impact sensory characteristics, making post‐fermentation methods the preferred choice for producing dealcoholized wines (Mangindaan et al., 2018). According to Regulation (EU) 2021/2117 (EP & CEU, 2021), Article 222b amends Annex VIII by incorporating dealcoholization processes, specifying physical separation techniques as the only permitted methods for post‐fermentation alcohol removal (Figure 1) despite encountering the same challenge of preserving the final product's quality (Mangindaan et al., 2018).

The complexity of alcoholic beverages has driven the adoption of advanced membrane and distillation technologies, enhancing efficiency and precision in production (Oro et al., 2025; X. Wang et al., 2024). These innovations are reshaping traditional practices by offering cleaner, more sustainable, and highly efficient solutions. The ability to isolate specific components at a molecular level has revolutionized winemaking and expanded into fields such as water treatment, food and beverage manufacturing, pharmaceuticals, and bioengineering. A defining characteristic of membrane systems is their high selectivity engineered to permit only specific molecules or ions to pass, determined by factors such as size, shape, charge, and chemical affinity (Oro et al., 2025).

Membrane‐based technologies are widely used for wine clarification and dealcoholization at both pre‐ and post‐fermentation stages. The choice of membrane type and material is crucial to preserving the sensory properties of wine. In pre‐fermentation, these methods help reduce sugar content in the must, while in post‐fermentation, they selectively remove alcohol to achieve the desired concentration in the final products. These applications highlight the adaptability and significance of membrane‐based technologies in modern winemaking practices (El Rayess et al., 2024). The primary technological challenge in lowering wine's alcohol content is removing ethanol while preserving its other components. Therefore, an effective strategy for producing low or dealcoholized wine must carefully balance ethanol removal, the chosen dealcoholization technique, energy consumption, and the potential impact on wine composition and sensory quality (Lisanti, Gambuti, & Moio, 2013; Varela et al., 2015).

This review provides a comprehensive examination of advances in physical techniques for wine dealcoholization, detailing various permitted methods used to produce dealcoholized wine. It explores how these techniques influence the chemical composition of wine and the sensory changes that occur because of alcohol removal. Additionally, it addresses the market potential across different regions, as well as stability challenges in preserving product quality and shelf‐life. Finally, the review concludes by reflecting on the prospects of dealcoholized wines, offering insights into the evolving landscape of this growing industry.

## 2 POST‐FERMENTATION TECHNOLOGIES FOR WINES DEALCOHOLIZATION

The reduction of alcohol content in wines may have two main objectives:

1. To achieve a better balance in the final product (excessive alcohol can result in a “hot” or harsh sensation, overshadowing other flavors and characteristics) (Schmitt & Christmann, 2022).
2. Consumers demand to access new low‐alcohol beverages while retaining the original sensory properties (Shubham & Shubhangi, 2024).

Wines with higher ethanol levels (15% (v/v)) are associated with increased bitterness, viscosity, and a velvety texture, whereas at higher pH values (3.4–3.6), these wines also exhibit enhanced heat sensation (Demiglio & Pickering, 2008; Demiglio et al., 2002). The rising demand for dealcoholized beverages is being driven by shifting consumer preferences and advancement in the dealcoholized industry (Kozłowski et al., 2021). Health‐conscious trends especially among the young generation have risen with many aiming to reduce disease risks, manage weight, or adopt a more mindful approach to alcohol consumption due to increasing awareness of mental health (Waehning & Wells, 2024).

Moreover, the phenolic compounds present in wine can be assimilated and undergo significant metabolism within the human body (Ferraz da Costa et al., 2020; Nemzer et al., 2022; Rodriquez‐Saavedra et al., 2023) and the consumption of beer and wine has been associated with various advantageous effects, including anti‐mutagenic and anti‐carcinogenic properties, cardioprotective benefits, immunomodulatory effects, and anti‐osteoporotic effects (Wurz, 2019). While wine is associated with numerous health benefits, the presence of alcohol can have negative effects on health, such as an increased risk of chronic diseases and adverse impacts on mental well‐being (Karunarathna et al., 2024). Therefore, removing or reducing alcohol from regular wine offers a promising solution by providing dealcoholized wine with reduced or no alcohol content. This allows consumers to enjoy the beneficial properties of wine without the potential adverse effects of alcohol (Hrelia et al., 2022), aligning with the preferences of health‐conscious individuals seeking mindful consumption options (Fuentes‐Fernández & del Campo‐Villares, 2025; Rai et al., 2025). It is evident that the separation of ethanol from beverages necessitates a technique of high selectivity, which can effectively isolate the alcohol component while minimizing any significant alteration to the quality features (Castro‐Muñoz, 2019a).

Some dealcoholization technologies not only remove ethanol but also extract a small amount of water, as reported in Table 2. This unintentional water loss leads to the concentration of other wine components, including polyphenols, flavonoids, and dry extract substances (Motta et al., 2017). In addition, the loss of water can influence the wine's sensory characteristics and can raise regulatory concerns regarding the type of water (endogenous or exogenous) that may be added to the final product.

**TABLE 2:** Predicted volume and water loss during total dealcoholization of wine using different technologies, highlighting alcohol removal efficiency and output alcohol concentration (data shared by VasonGroup).

Technology	Alcohol content % (v/v)	Alcohol removed % (v/v)	Output alcohol concentration % (v/v)	Total volume loss (%)	Total water loss (%)
Membrane contactor	10	9.5	Next to 100%	9.5	Nothing
Vacuum distillation (with rectification)	10	9.5	85	11.2	1.7
Vacuum evaporator	10	9.5	40	23.8	14.3
Spinning cone column	10	9.5	40	23.8	14.3
Pervaporation	10	9.5	40	23.8	14.3

Low‐alcohol and alcohol‐free wines (with no alcohol at all) can be obtained with different methods: (i) reverse osmosis, (ii) osmotic distillation, (iii) vacuum distillation, (iv) spinning cone column, (v) pervaporation, and (vi) diafiltration.

### 2.1 Reverse osmosis

**FIGURE 2:**
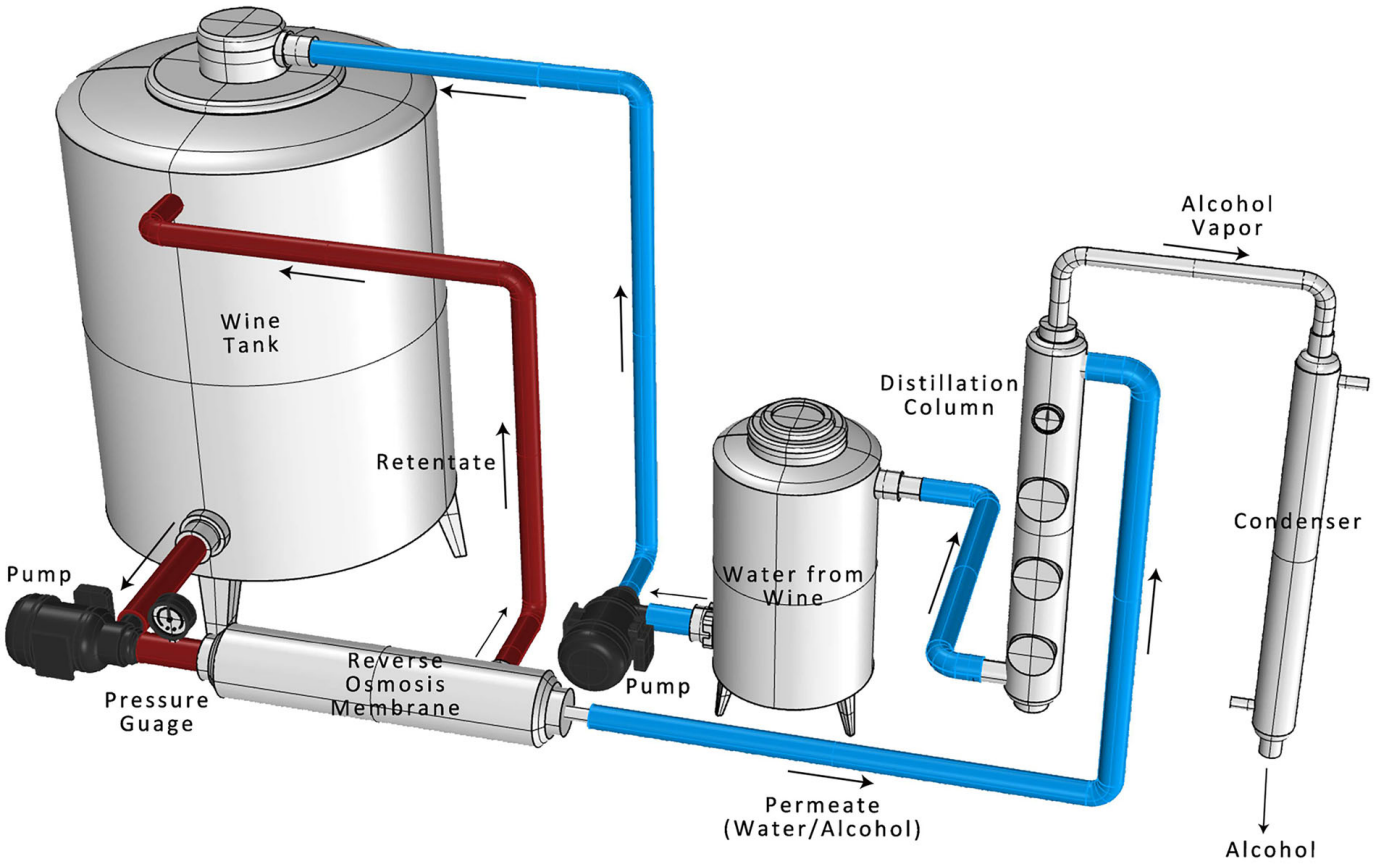
Schematic representation of the reverse osmosis process for wine dealcoholization, demonstrating the use of a semi‐permeable membrane under high pressure to selectively remove ethanol while preserving key wine constituents.

Reverse osmosis (RO) is a filtration method that employs a semipermeable membrane with a pore size of 0.1–1 nm, which retains molecules larger than a certain size to segregate alcohol from wine. The process of dealcoholization involves subjecting wine to high pressure, which forces it through a semipermeable membrane, leading to the separation of alcohol and other unwanted constituents, ultimately yielding a dealcoholized wine (Catarino & Mendes, 2011a; Catarino et al., 2007) (Figure 2). The concentration of each component, including alcohol, rises as a result of the process. The retentate must be restored with the original water removed to counteract this impact and obtain the required amount of ethanol reduction. To accomplish this, it is required to separate the water from the alcohol, which is accomplished by thermal distillation. The original water is reintroduced into the retentate, and the process yields a byproduct with a high ethanol concentration (Bogianchini et al., 2011; Pham et al., 2019).

The utilization of RO instead of conventional distillation offers several benefits including the production of high‐quality dealcoholized products comparable to standard alcoholic products, minimal degradation of temperature‐sensitive compounds, and reduction of the volatile acidity. This is due to the low‐temperature nature of the process and the absence of phase change for alcohol removal, which mitigates chemical alteration and physical losses (Catarino et al., 2007). However, energy consumption for RO is significant because of the need to generate high pressure, and the installation cost might be considerable because of the large membrane area necessary for the desired output. Owing to the high production cost involved in generating high pressure, forward osmosis (FO) could be another method for wine dealcoholization, which is discussed in a subsection (2.1.1) below.

Pilipovik and Riverol (2005) assessed the delocalization system based on RO to determine the feed flux through the membrane by Equation (1).

(1) $$J_{w}=\left[P_{f}-\frac{\Delta P_{d}}{2}-P_{P}-\frac{P_{osf}+P_{osb}}{2}\right]k_{w}\left(\frac{5}{d}\right).$$

However, the osmotic pressure is given by $P_{osf}=0.081T\sum m_{i}$. The solute flow is determined by applying Equation (2).

(2) $$J_{s}=k_{s}\;\left(\Delta C\right)\left(\frac{S}{d}\right),$$

*J_w_* is the permeate production, *P_f_* is the feed pressure, *P_d_* is the difference between feed pressure and permeate pressure, *P_p_* is the permeate pressure, *P_osf_* is the osmotic pressure of the feed, *P_osb_* is the osmotic pressure of the brine, *K_w_* is the membrane permeability coefficient for the permeate, *T* is the temperature, *m* is the molar concentration of all constituents of the solution, *J_s_* is the solute rate, *K_s_* is the membrane permeability coefficient for alcohol, *S* is the membrane surface area, and *d* is the membrane thickness.

In another study, Catarino and Mendes (2011a) measured the solvent flux (*J_A_*) of the RO membrane using Equation (3).

(3) $$J_{A}=k_{A}\;\left(\Delta P-\Delta \pi \right).$$

However, the selectivity of RO membranes can be quantified based on the rejection or retention coefficient (*R*).

(4) $$R_{i}=1-\frac{C_{p,i}}{C_{r,i}}.$$

Furthermore, two other equations were developed to calculate ethanol history concentration in the retentate (dealcoholized wine) as a function of the diafiltration time (Equation 5) and the amount of water added to the retentate to balance the permeating flux (Equation 6).

(5) $$C_{r,E}\left(t\right)=C_{f,E}\;\exp\left[-\frac{Q_{w}t}{V_{f}}\left(1-R\right)\right],$$

(6) $$C_{r,E}\left(V_{w}\right)=C_{f,E}\;\exp\left[-\frac{V_{w}}{V_{f}}\left(1-R\right)\right],$$

where *k_A_* represents the permeance toward solvents (known as hydrodynamic permeability coefficient), *ΔP* reflects the pressure difference between both sides of the membrane, and *Δπ* is the osmotic pressure. The concentrations of compound *i* in the permeate and retentate sides are represented as *C_p,i_* and *C_r,i_*_,_ respectively. C_r,E_ represents the concentrations of ethanol on the retentate side where C_f,E_ is the ethanol concentration in the original feed solution. Q_w_ is denoted as the flow rate of water. V_f_ is the feed solution volume, and t is the permeation time. V_w_ is the total volume of water required in a diafiltration time. *R* is the retention or rejection coefficient.

A combined treatment of RO with osmotic distillation (OD) was applied to partially dealcoholize Australian Shiraz wine (16%–17% (v/v)), reducing alcohol to 13.5% (v/v) and 10.5% (v/v). While basic wine parameters (malic acid, acetic acid, glycerol, TA, and pH) remained stable, the volatile profile changed significantly, with esters decreasing by nearly 50% at 10.5% (v/v) due to their high volatility and affinity for the hydrophobic membrane. Sensory analysis showed minimal differences at 13.5% (v/v), but at 10.5% (v/v), dark fruit, raisin/prune, and black pepper attributes diminished, while herbaceous perception increased, with lower astringency and overall aroma intensity (Longo et al., 2018b). These findings suggest that RO‐OD effectively reduces alcohol while preserving structure, but excessive dealcoholization (>10.5% (v/v)) compromises sensory quality. Similarly, Pham et al. (2020) also applied combined reverse osmosis‐evaporation pertraction (RO‐EP) process on five Cabernet Sauvignon wines to partially dealcoholize them to remove the alcohol by −1.3 to −2.5% (v/v) at industrial scale. The impact of the RO‐EP treatment during the sensory analysis on wine fragrance and flavor was found to be not significant, aligning with the limited compositional alterations found between original and partially dealcoholized wine. Only five aroma attributes, namely red fruit, black fruit, green, confectionery, and chocolate were found to have significant differences in the intensity rating of wines; however, there were no significant variances in the total intensity of wine aroma observed (Pham et al., 2020).

Many sensory characteristics commonly used by wine specialists, including length in the mouth, finish, persistence, and aftertaste, emphasize the temporality of the sensations experienced during tasting (Meillon et al., 2009). Meillon et al. (2010) employed RO to remove alcohol from red wines, namely Syrah, resulting in three different levels of alcohol reduction: 2% (v/v), 4% (v/v), and 5.5% (v/v). The sensory result by temporal dominance analysis revealed that as the alcohol content declined, there was an observed increase in the sense of astringency, while the sensation of heat was diminished (Meillon et al., 2010). The authors suggest that ethanol's heat sensation influences the perception of astringency by interacting with the trigeminal system. Recent studies have shown that ethanol reduces salivary protein–tannin interactions in both model solutions and red wine, supporting this effect (Gambuti et al., 2011; Rinaldi et al., 2012).

Another study by  Meillon et al. (2009) investigated the standard wine and RO dealcoholized wine with −3% (v/v) and −1.5% (v/v) alcohol reduction, and reconstituted wine of both Syrah and Merlot varieties. The standard Merlot had pronounced sensations of heat and bitterness, while the standard Syrah displayed a dominant acidic profile, transitioning to a noticeable heat sensation onward, and concluding with a lingering bitter taste. The Merlot wine with a reduced alcohol content of −3% (v/v) exhibits notable astringent and heat characteristics. Similarly, Syrah wine with a similar reduction in alcohol content also displays significant acidity, red fruit flavors, bitterness as well as astringency. This finding aligns with a previous study that observed an increase in perceived astringency with a decrease in alcohol content, as described earlier (Meillon et al., 2010). The Merlot wine −1.5% (v/v) dealcoholization was found to have bitter, heat, and astringent qualities. Specifically, the astringent sensation was the most prominent, followed by bitter and red fruit and heat sensation. While Syrah −1.5% (v/v) exhibited a notable decrease in its overall sensory profile, primarily characterized by a pronounced bitter feeling. When considering reconstituted Merlot, it was initially perceived as a bitter taste, followed by a subsequent sensation of heat. The conclusion of this wine was distinguished by the simultaneous presence of bitter and astringent perceptions. In contrast, the reconstituted Syrah exhibited notable characteristics of woodiness, astringency, bitterness, and heat (Meillon et al., 2009).

Gil et al. (2013) applied RO to Cabernet Sauvignon (14.8% (v/v)) and Grenache‐Carignan (16.2% (v/v)) wines, reducing alcohol by −1% (v/v) and −2% (v/v). The process had no significant impact on pH, color, total phenolics, or proanthocyanidins, with only a slight increase in titratable acidity and minor anthocyanin loss in −2% (v/v) Grenache‐Carignan. Polysaccharides remained stable at −1% (v/v) but increased slightly at −2% (v/v), likely due to reduced ethanol‐induced precipitation. Sensory analysis showed minimal perceptible differences, with Grenache‐Carignan (−2% (v/v)) distinguishable, when served at 24–26°C (Gil et al., 2013). These results confirm that RO enables moderate alcohol reduction while preserving wine composition and sensory quality, making it an effective technique for balancing ethanol content.

#### 2.1.1 Forward osmosis

FO is a membrane‐based technique widely used in water treatment (J. Wang & Liu, 2021) and food processing specifically in juice concentration (Tavares et al., 2022; H. Wang et al., 2022; Zhang et al., 2021), and it has been explored for ethanol separation as a non‐thermal method to reduce the alcohol content (Ambrosi et al., 2017). Research on the use of FO for wine dealcoholization is still in the developmental stage, with only a fewer studies reported in terms of dealcoholization. FO utilizes the semipermeable membrane (like in RO) to separate the components based on their size and properties and uses a draw solution with high osmotic pressure that creates a concentration gradient across a semi‐permeable membrane, causing water and alcohol from the feed to move through the membrane and dilute the draw solution (Ambrosi et al., 2020). Forward osmosis typically utilizes membranes made from integrated cellulose acetate structures or thin‐film composite aromatic polyamides. These membranes share similar chemical compositions and structural properties with those used in RO and nanofiltration. Unlike pressure‐driven membrane processes, FO offers some benefits, such as operating at low pressures and potentially being more cost‐effective (Ambrosi et al., 2018).

However, only a limited number of studies are reported on using FO for wine dealcoholization. One such study reported by Huang et al. (2022) provides valuable insights into the application of FO in wine dealcoholization, where kiwi wine was dealcoholized using cellulose triacetate membrane using three types of draw solutions (NaCl, MgCl_2_, KCl). The dealcoholized kiwi wine using MgCl_2_ as the draw solution was found to be optimal due to its high‐water flux (11.1 L/m^2^ h), minimum membrane fouling, and preserving the highest content of volatiles (2735.36 µg/mL) and phenolic (91.59 µg/mL) compounds with alcohol content of 0.45% (v/v) in dealcoholized wine (Huang et al., 2022).

### 2.2 Osmotic distillation

**FIGURE 3:**
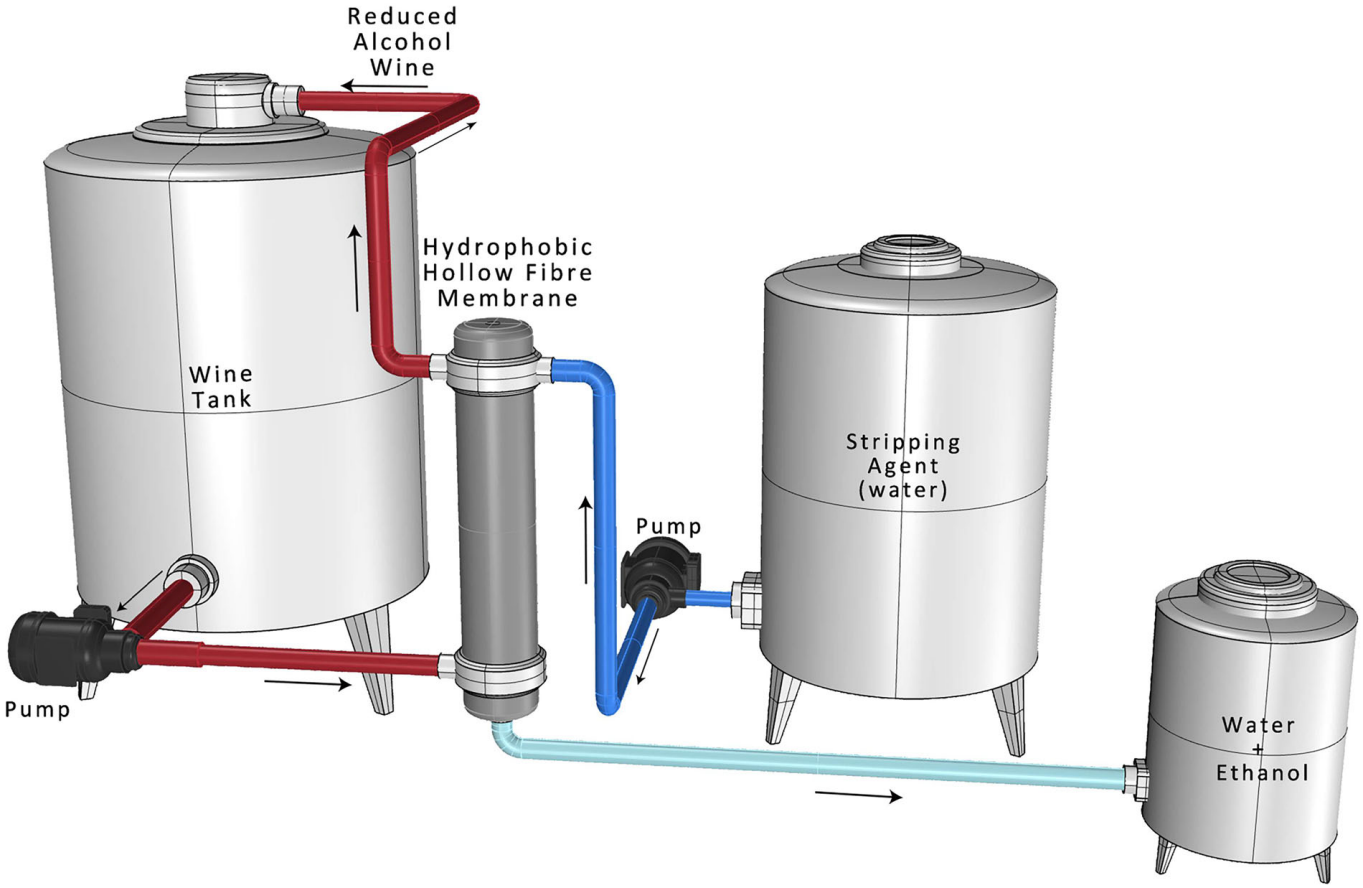
Schematic representation of the osmotic distillation process for wine dealcoholization, illustrating the use of a hydrophobic, microporous membrane to facilitate the selective removal of ethanol through a vapor pressure gradient, while preserving the wine's aromatic and sensory properties.

There has been a growing interest in osmotic distillation (OD) as a potential method for producing wines with reduced alcohol content (Esteras‐Saz et al., 2021). The process of osmotic distillation involves the employment of a hydrophobic hollow fiber membrane contactor that separates the wine and ethanol extracting agent (stripper) without the application of heat. The hydrophobic membrane's porous matrix allows ethanol, the process's principal volatile component with a high vapor pressure, to transition from the liquid to the gaseous phase. Water is typically used as the stripping phase, ensuring no dangerous byproducts (i.e., extractants), and the procedure is simple, safe, and performed at ambient temperature and atmospheric pressure, saving energy and minimizing the loss of volatile compounds. Alcohol removal through OD is typically applied to alcoholic beverages such as wine and beer (Gryta, 2018; Loredana et al., 2018; Nagaraj et al., 2006; Russo et al., 2019). The technique did not decrease the water content in the processed wine, and the loss in product is only due to the alcohol removal. Figure 3 illustrates the osmotic distillation process for wine dealcoholization.

Several mathematical models and equations have been reported to calculate the performance of the OD process. According to Varavuth et al. (2009), the driving force corresponds to the bulk concentration of both sides of the OD membrane, and the flux is described by Equation (7).

(7) $$J_{e}=k_{ov}\;\Delta P_{b},$$

where *J_e_* represents the ethanol flux, *ΔP_b_* (*P_ef,b_* − *P_es,b_*) is the ethanol vapor difference between the feed and stripping sides, and *K_ov_* is denoted as the overall mass transfer coefficient, which can be obtained by $\frac{1}{k_{ov}}=\frac{1}{k_{f}}+\frac{1}{k_{m}}+\frac{1}{k_{s}}$, where *k_f_*, *k_m_*, and *k_s_* represent the mass transfer coefficients of the feed boundary layer, membrane, and stripping boundary layer, respectively. Esteras‐Saz et al. (2021) and Esteras‐Saz et al. (2024) determined ethanol flux through the OD membrane at set time intervals using ethanol concentration and stripping weight by Equation (8).

(8) $$J_{Exp}=\frac{\Delta W}{A_{e}\Delta t}.\;$$

The use of fixed time intervals calculations was performed because the driving force across the membrane decreases with time in a recycle mode operation, which in turn affects the ethanol flux across the membrane. Δ*W* is the variation of ethanol amount in the feed or stripping at the specific interval of time *ΔT* and A_e_ overall effective membrane contact area (Esteras‐Saz et al., 2021). The loss of volatile organic compounds from wine via the membrane appears to be correlated with Henry's constant values, according to recent studies (Diban et al., 2013, 2008). Thus, in OD, the flow of each volatile component over the membrane, measured in *J^i^* (gm^−2^s^−1^), could be represented as:

(9) $$J^{i}=K_{G}^{i}\;\left(\frac{\rho_{f}H_{f}^{i}}{M^{i}}C_{f}^{i}-\frac{\rho_{s}H_{s}^{i}}{M^{i}}C_{s}^{i}\right),$$

where $K_{G}^{i}$ is denoted as the global mass transport coefficient, *ρƒ* and *ρs* (kg m^−3^) are the molar density in the feed and the stripping side, respectively. *M^i^* is the molar weight (g mol^−1^) of component *i*, $H_{f}^{i}$ and $H_{s}^{i}$ are the Henry constant (Pa m^3^ mol^−1^) of component *i* in feed and stripping side, respectively. $C_{f}^{i}$ and $C_{s}^{i}$ are the components *i* concentrations in the feed and stripping side, respectively (Esteras‐Saz et al., 2021).

The loss of volatile molecules during dealcoholization with membrane contactors is a very complicated process that involves several chemical–physical equilibria. A significant percentage of generic volatile compounds, such as ethanol, is lost through pertraction (Varavuth et al., 2009). In addition, 2%–3% loss of aroma could be attributed to adsorption on the membrane (Diban et al., 2008), which can be calculated by Equation (10).

(10) $$\mathrm{Aroma}\;\mathrm{loss}\;=\frac{{\left[\mathrm{aroma}\right]}_{0}-{\left[\mathrm{aroma}\right]}_{F}}{{\left[\mathrm{arom}\alpha \right]}_{0}}\times 100,$$

where [aroma]_o_ is the initial and [aroma]_F_ is the final concentration of the specified aroma (Esteras‐Saz et al., 2024).

A study reported on dealcoholized white wine from an ancient Italian grape variety (cv Falanghina, 12.5% (v/v)). The wine was dealcoholized eight times with each cycle lasting for 30 min, reducing the alcohol content by 30% of its volume. The primary quality attributes of dealcoholized wine samples were analyzed following alcohol reduction to levels ranging from 9.8% to 0.3% (v/v). Wines with varying alcohol concentrations exhibited comparable total phenolic content, flavonoids, organic acids, and titratable acidity, with statistical significance (*p* < 0.05). However, alcohol removal substantially impacted the volatile compound profile. Specifically, only 50% of higher alcohols, acids, and lactones were retained at an alcohol concentration of 9.8% (v/v), ∼30% at 6.8% (v/v), and progressively lower levels were preserved in samples with reduced alcohol content with an ultimate imbalance in taste profile (Liguori et al., 2019). Corona et al. (2019) optimized wine and water flow rate ratios to efficiently reduce alcohol content with minimal water use, lowering Montepulciano wine from 13.2% (v/v) to 2.7% (v/v) over five dealcoholization cycles. Residual sugar slightly increased, softening acidity perception due to reduced alcohol. The dealcoholized samples showed variations in acidity and pH, while color, anthocyanins, flavonoids, and phenols remained largely unchanged. While dealcoholization led to a decrease in the perception of red fruits, spices, sweetness, bitterness, and astringency, acidity perception increased due to the reduced masking effect of ethanol. However, sensory acceptability remained satisfactory up to an alcohol reduction of −7.8% (v/v), corresponding to a final ethanol content of 5.4% (v/v) (Corona et al., 2019).

OD was applied for partial dealcoholization of Montepulciano d'Abruzzo wine (13.2% (v/v)), reducing alcohol to 8.31% (v/v), 6.95% (v/v), and 5.41% (v/v). While OD preserved total acidity, tartaric acid, and pH, progressive alcohol reduction led to significant volatile losses, particularly ethyl hexanoate, ethyl octanoate, and isoamyl acetate, diminishing aromatic complexity (Russo et al., 2019). In another study, partial dealcoholization of Aglianico red wine (15.37% (v/v) and 13.28% (v/v) at −2%, −3%, and −5% (v/v) induced notable sensory and chemical modifications. While moderate dealcoholization (−2% (v/v)) preserved the sensory profile, higher reductions (−5% (v/v)) resulted in a significant loss of red fruit, cherry, and spicy aromas, coupled with an increase in astringency and acidity. The volatile profile was notably affected, with a marked reduction in esters and higher alcohols, particularly isoamyl acetate and ethyl hexanoate (up to 60%), leading to diminished fruitiness. However, color stability, total flavonoids, and phenolic content remained unchanged. Sensory evaluation indicated that wines subjected to −5% (v/v) dealcoholization exhibited lower acceptability with a more astringent taste profile, emphasizing the adverse impact of excessive ethanol removal on overall wine quality (Lisanti, Gambuti, Genovese, et al., 2013). This finding aligns with earlier studies that have demonstrated the inhibitory impact of ethanol on the perceived intensity of astringency (Fontoin et al., 2008).

OD is a promising technique for wine dealcoholization, particularly for moderate ethanol reductions, as it preserves key phenolic compounds, acidity, and color. However, its effectiveness is limited by the progressive loss of volatile compounds, which compromises sensory perception, especially at higher levels of alcohol removal, necessitating process optimization and aroma recovery strategies to maintain wine balance and enhance sensory quality.

### 2.3 Vacuum distillation

The process of vacuum distillation (VD) involves the removal of alcohol from wines through the process of evaporation followed by condensation. VD is performed in a vacuum where alcohol is separated from wine based on its boiling point under reduced pressure. The application of vacuum in the process of ethanol distillation results in a reduction of the evaporation temperature by 15–20°C. Additionally, the initial distillate fractions are retrieved and subsequently combined with the dealcoholized fraction toward the end of the treatment. The reduction of volatile compound losses can be achieved through the recovery of the initial fractions of distillate, which are rich in aromatic compounds, and the use of a low operating temperature (Andrés‐Iglesias et al., 2015; Motta et al., 2017).

**FIGURE 4:**
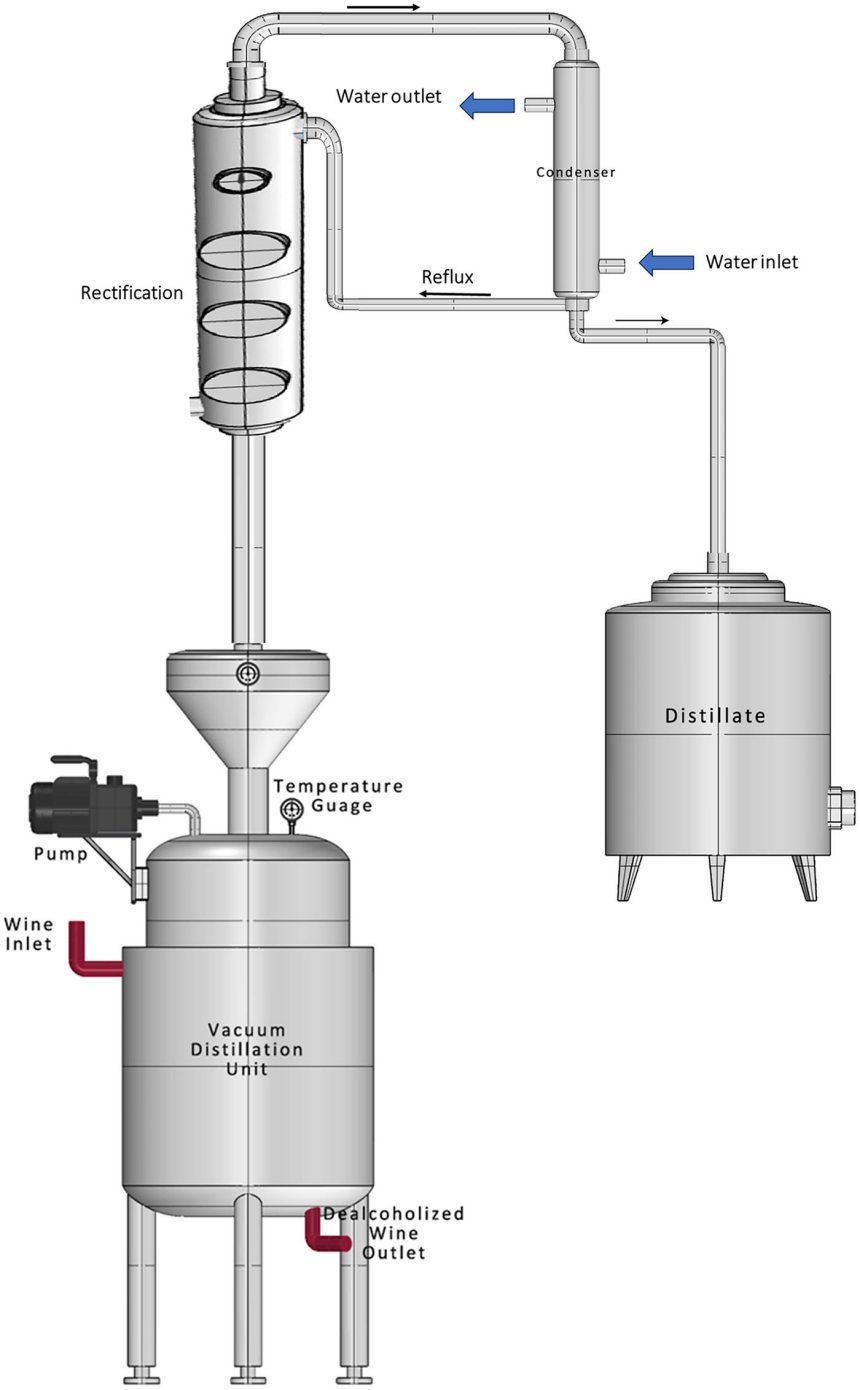
Illustration of the vacuum distillation process for wine dealcoholization, highlighting the role of low‐pressure conditions in facilitating ethanol evaporation at reduced temperatures.

It is noteworthy that the implementation of VD technology can vary based on the requirements of the winemaker or the targeted degree of alcohol reduction, thereby necessitating the use of distinct apparatuses and configurations. The technologies that could be employed in this context encompass vacuum flash evaporation, vacuum rectification, or hybrid approaches that integrate VD with techniques such as cold extraction (Motta et al., 2017; Schmitt & Christmann, 2022). Industries requiring accurate component separation at lower temperatures frequently employ VD technology (Labrado et al., 2020; X. Wang et al., 2024). Vacuum distillation leads to a partial evaporation of endogenous water along with alcohol, for which it is important to provide a rectification column to recover a part of it (Table 2). VD does have the advantage of removing alcohol to a controllable extent, but it also significantly affects the physicochemical parameters resulting in wine with poor sensory profile. Figure 4 shows the typical VD process for dealcoholizing wine which can be modified with the addition of a rectification column to recover the endogenous water. The latest study by Veiga‐del‐Baño et al. (2024) explores GoLo, an advanced vacuum distillation technology that completely removes alcohol from wine (<0.1% (v/v)) while preserving its chemical profile of wine. The technique was tested on white, rosé, and red Spanish wines, and the study confirms 100% ethanol removal, validating GoLo's effectiveness and its potential for producing high‐quality alcohol‐free wines.

Petrozziello et al. (2019) employed VD to dealcoholize Chardonnay wine (10.79% (v/v)), reducing ethanol content by −7.43% (v/v) to 3.36% (v/v), with a focus on volatile compound retention. The process had a minimal impact on non‐volatile components such as acids, salts, and polyphenols but led to substantial volatile losses including 3‐methyl‐1‐butanol acetate, ethyl hexanoate, ethyl octanoate, hexyl acetate, 1‐hexanol, and other 6‐carbon alcohols were no longer quantifiable or even measurable in the wine after being dealcoholized at 3.36% (v/v). Sensory evaluation revealed a decrease in aromatic complexity, lower ethanol heat perception, and an imbalance in mouthfeel, emphasizing the pronounced effects of extensive ethanol removal on wine quality (Petrozziello et al., 2019). Sam, Ma, Liang, et al. (2021) applied VD to Chardonnay (13.4% (v/v)), Pinot Noir rosé (12.2% (v/v)), and Merlot (13.9% (v/v)), reducing alcohol content to 0.7% (v/v). The process significantly altered the chemical and sensory profile of the wines. VD caused a significant increase in total acidity and a reduction in SO_2_ levels, which could influence the wine's oxidative stability. The dealcoholization process resulted in an 85%–98% decrease in volatile compounds, particularly esters, higher alcohols, and terpenes, leading to a loss of fruitiness and floral aromas. Sensory analysis revealed a decline in aroma intensity, viscosity, and sweetness, while the perception of acidity was heightened, ultimately lowering overall acceptability (Sam, Ma, Liang, et al., 2021).

Another study also reported a significant loss of volatile compounds, particularly higher alcohols and esters, impacting the fruity and fresh sensory attributes when VD was used to dealcoholize Langhe Rosé (13.2% (v/v)), Verduno Pelaverga (15.2% (v/v)), and Barbera (14.6% (v/v)) wines to 5% (v/v) (Motta et al., 2017). Similarly, the reduction of alcohol in Isabella dry red wine from 9.8% (v/v) to 0.4% (v/v) led to a significant shift in its chemical profile, with a decline in higher alcohols, acetaldehyde, and esters, affecting the wine's aromatic depth. To counteract these sensory changes, researchers added grape must cryoconcentrate to restore sweetness and balance acidity. According to the authors, this adjustment helped mask the wine's sharper acidity and enhanced the overall harmony of the dealcoholized wine (Uspalenko et al., 2024). The reported studies indicate that while vacuum distillation effectively reduces ethanol, it also leads to significant volatile compound losses, increasing acidity and reducing fruitiness, which compromises the wine's sensory balance.

### 2.4 Spinning cone column

Spinning cone column (SCC) technology has found widespread application in the food industry, particularly for the recovery of coffee and tea aromas, as well as for the extraction of volatile oils (Riley & Sykes, 2002). SCC is used to dealcoholize wine as it is engineered to almost selectively remove alcohol from wine, without affecting the wine's flavor and aroma. The SCC is efficient for heat‐sensitive items like wine since it operates based on molecular distillation. The employment of the SCC method has the potential to decrease the ethanol content from 15% (v/v) to below 1% (v/v) (Brányik et al., 2012). Also, the distillate, which is a byproduct of dealcoholizing wine and beer, can be used to create aromatic spirits (Takács et al., 2007).

**FIGURE 5:**
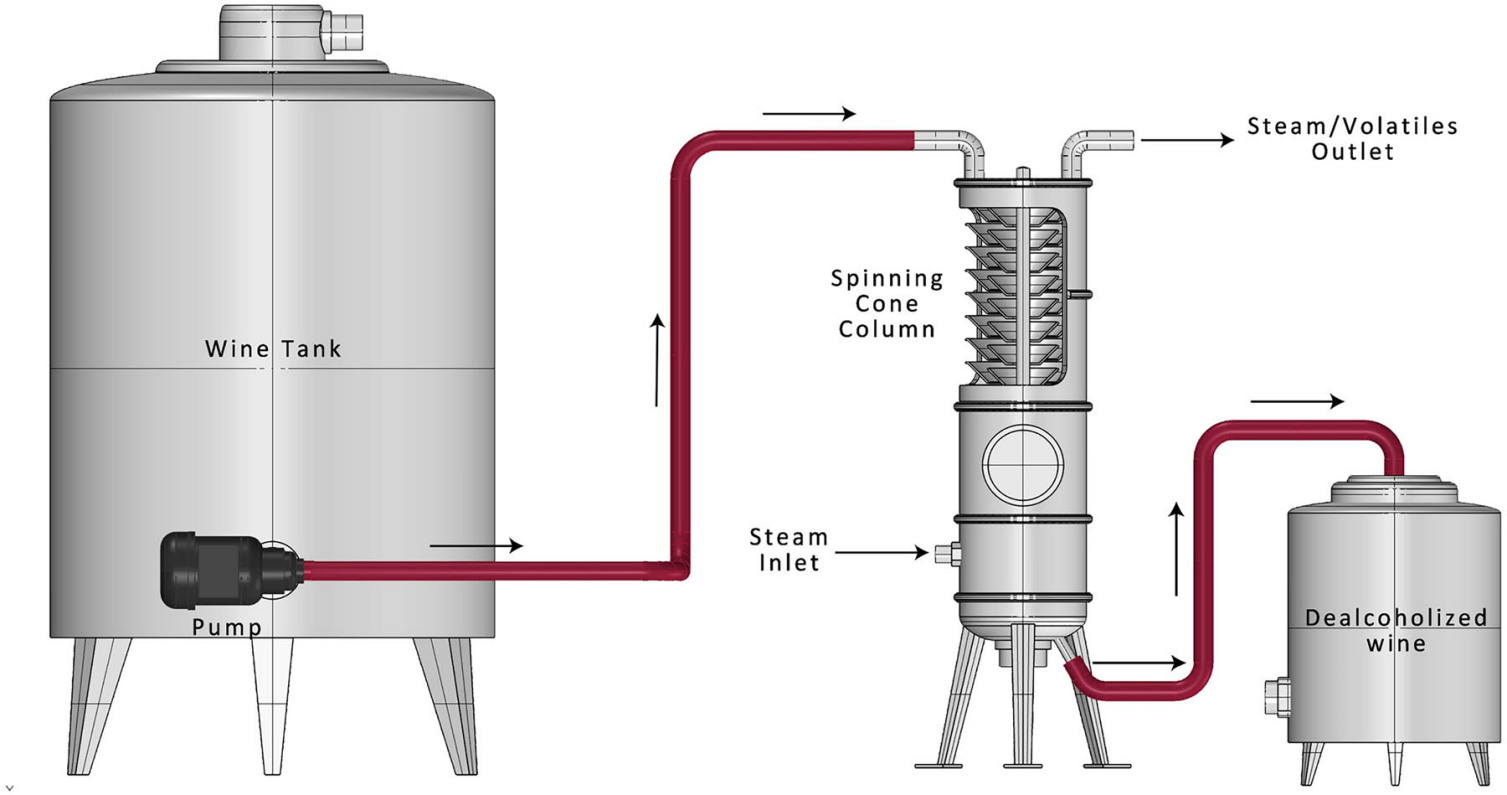
Schematic overview of the spinning cone column dealcoholization method, highlighting the role of vacuum and rotating cones in efficiently removing ethanol while ensuring the preservation of desirable aroma compounds.

SCC consists of a vertical column made of a stainless‐steel vessel with alternating fixed cones attached to the inner wall and spinning cones attached to the shaft that rotates in the middle of the column (Figure 5). Liquid poured into the top of the column runs down the first stationary cone forming a thin film and into the base of the first spinning cone due to gravity. The liquid is subjected to centrifugal force in upward and outward directions across the surface of the rotating cone forming a thin film. Subsequently, the liquid is propelled into the air and descends onto the subsequent stationary cone. Since this process is repeated, the liquid eventually moves downward. At the same time, a counter‐current (upward) flow is produced by injecting steam from the base of the column. As steam moves over the surface of the thin films and combines with airborne liquid droplets, volatile chemicals are stripped into the vapor. Fins attached to the bottom of each rotating cone generate turbulence in the liquid and vapor phases, hence accelerating the mass‐transfer rate. The stripped liquid remaining at the bottom of the column is recovered, while the volatile‐enriched vapor flowing out the top of the column is sent, re‐condensed, and recovered as a concentrated liquid form. In general, the rotating cone column technology provides a controlled and effective method to dealcoholize wine. By meticulously controlling temperature, vacuum, and the formation of a liquid film, SCC enables the removal of alcohol while preserving the wine's desirable sensory qualities (Khonsha et al., 2013; Puglisi et al., 2022).

Belisario‐Sánchez et al. (2012) did not use external steam as a stripping agent; instead, they used the wine (about 1%) that was discharged from the column and transformed into a low‐temperature vapor state due to the flash evaporation in the high‐vacuum setting within the column. The cold steam functioned as a stripping agent. The process of dealcoholization of wine can be carried out in two distinct stages, namely the initial stage of aroma recovery at a temperature of 26°C, followed by a subsequent stage of ethanol removal at a temperature of 40–50°C. Following the separation of ethanol, the entirety of the aromatic fraction recovered at the initial stage can be reintroduced into the wine (Belisario‐Sánchez et al., 2012; Huerta‐Pérez & Pérez‐Correa, 2018). High ethanol recoveries can be achieved by running SCC at moderate temperatures, medium‐to‐high stripping rates, and medium‐to‐high liquid flow rates. Low temperatures, intermediate stripping rates, and high liquid flow rates are optimal for SCC operation, leading to high ethanol concentrations in the distillate (Huerta‐Pérez & Pérez‐Correa, 2018). The byproduct derived from the dealcoholization of wine and beer, known as distillate, possesses significant value as a resource to produce fragrant spirits (Takács et al., 2007). The unified process of SCC dealcoholization and the valorization of the distillate results in a reduced environmental impact and a decrease in the consumption of natural resources (Margallo et al., 2015). In case of dealcoholization below 0.5% (v/v), the processed wine will be decreased in volume to 25% due to the loss of endogenous water. To calculate the ethanol recovery (*Re_ETOH_*) obtained in SCC dealcoholizing plant at lab scale, Huerta‐Pérez and Pérez‐Correa (2018) used Equation (11).

(11) $$Re_{ETOH}=\frac{D\cdot w_{D}}{L\cdot w_{L}},$$

where *D* represents the total mass of the distillate, *w_D_* is the ethanol mass fraction in the distillate. *L* is the mass of liquid/wine poured in the SCC column, and *w_L_* is the mass of ethanol fraction in the liquid/wine poured in the column.

Belisario‐Sánchez et al. (2009) analyzed the phenolic composition of 19 different wines before and after dealcoholization using SCC, finding that most phenolic compounds, including resveratrol, flavonols, and anthocyanins, remained stable or slightly increased due to the concentration effect following ethanol removal. However, a slight reduction in antioxidant activity was observed, primarily attributed to SO₂ loss, which plays a role in radical scavenging capacity. In another study, Belisario‐Sánchez et al. (2012) examined aromatic‐enriched dealcoholized wine produced via SCC distillation, where Tempranillo (red), Cabernet Sauvignon (rosé), and Chardonnay (white) wines, initially at 14% (v/v) alcohol, were dealcoholized to 0.5% (v/v), and their separated aromatic fraction was reintroduced to restore the sensory profile. Chemical analysis indicated that the ethanol removal process led to a concentration of certain aromatic compounds, while sensory evaluations revealed no significant differences between the raw and dealcoholized wines, with some samples also exhibiting enhanced aromatic attributes.

R. L. García et al. (2021) evaluated the impact of SCC dealcoholization on Listán Prieto wines, reducing their alcohol content to 6.0% (v/v) and 0.5% (v/v). The results showed that SCC‐treated wines exhibited higher acidity, particularly at 6.0% (v/v), making them the least preferred among tested samples. Wines dealcoholized to 0.5% (v/v) were associated with bitterness, astringency, and red fruit aromas, but their overall acceptability was low. Compared to another method (RO) used in the same study, SCC wines had reduced sweetness and body, leading to lower hedonic ratings in sensory analysis (R. L. García et al., 2021). In another study, trained panelists detected changes in aroma intensity and hot mouthfeel with a 0.4% (v/v) difference in a Chardonnay wine (14.9% (v/v)) that underwent partial dealcoholization using SCC technology and was blended in 0.2% (v/v) increments down to 12.9% (v/v). However, consumers did not perceive differences below 1% (v/v). Consumer preference remained consistent across the different alcohol levels evaluated, suggesting that a 2% (v/v) reduction in Chardonnay has minimal impact on sensory perception and liking, supporting the feasibility of partial dealcoholization (King & Heymann, 2014). In a unique study, Puglisi et al. (2022) investigated the removal of smoke taint from wine produced from smoke‐affected grapes. However, SCC distillation proved ineffective, as volatile phenols remained largely unchanged, while their glycoconjugates became more concentrated. Additionally, the removal of desirable volatile aroma compounds further intensified the perception of smoke‐related sensory attributes instead of reducing them. SCC is an effective technique for retaining phenolic compounds and certain aromatic fractions, particularly when aroma recovery steps are applied. However, its impact on sensory perception depends on the degree of alcohol removal, with higher reductions often leading to imbalances in acidity, sweetness, and body. Optimizing the process is crucial to improving the sensory quality of fully dealcoholized wines and minimizing undesirable changes in aroma and mouthfeel.

### 2.5 Pervaporation

**FIGURE 6:**
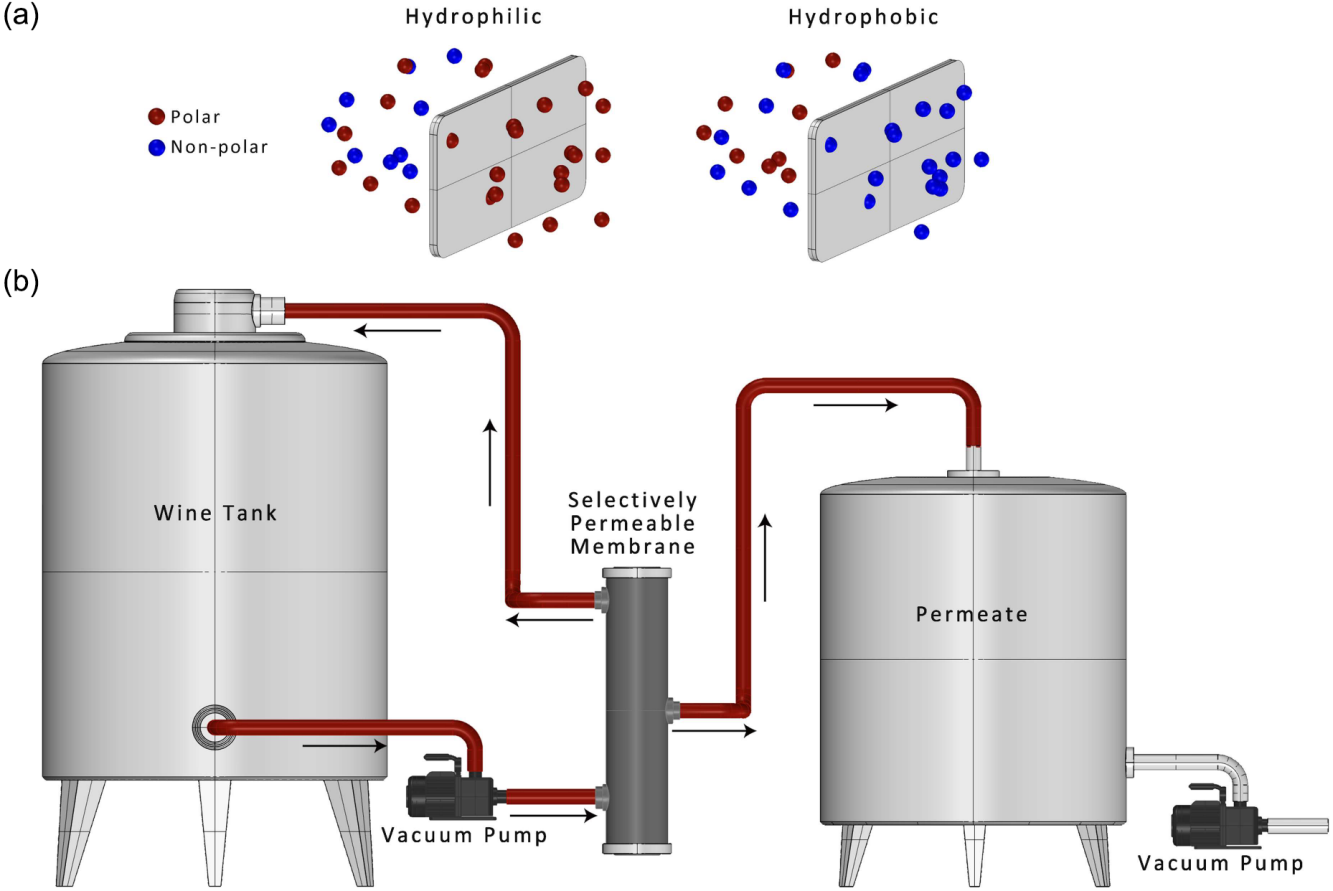
(a) Illustration of hydrophilic and hydrophobic membrane and (b) schematic overview of the pervaporation process for wine dealcoholization, highlighting the role of selective membrane transport in removing ethanol while maintaining wine aroma and flavor integrity.

Pervaporation is a separation methodology illustrated in Figure 6b that involves the partial vaporization of a liquid feed through a selectively permeable membrane that is nonporous. A crucial element of pervaporation involves the transfer of phases for the diffusing compounds from the feed stream to the permeate stream (Camera‐Roda et al., 2018). The feed mixture is directed to one side of the membrane, and a fraction of the feed undergoes vaporization as it permeates through the membrane to the opposite side. The permeate is then extracted using a carrier gas or vacuum and subsequently condensed into a liquid state. The process of mass transfer in pervaporation is composed of a sequence of three successive stages: (i) selective feed side membrane absorption, (ii) membrane selective diffusion, and (iii) permeate‐side carrier gas desorption (Castro‐Muñoz, 2019a; Castro‐Muñoz et al., 2023; Jyoti et al., 2015; Koros & Zhang, 2017). The pervaporation membrane is characterized by its non‐porous nature and can be of hydrophilic or hydrophobic/organophilic properties as illustrated in Figure 6a (Castro‐Muñoz, 2019b), which are contingent upon the specific material utilized in its construction (Castro‐Muñoz et al., 2023; Liu & Jin, 2021; Takács et al., 2007).

The simplest way to explain pervaporation is through the solution‐diffusion model. The pervaporation process is usually described by the total flux (*J*) of the component that passes through the membrane and the separation factor (β) of one component relative to another (Sun et al., 2020). Sun et al. (2020) calculated the partial flux (*Ji*) of component *i* through the membrane, which can be obtained using Equation (12).

(12) $$J_{i}=\frac{\Delta mi}{A\Delta t},$$

where *Δmi* represents the mass of the component *i* (g) over the specific interval of time *Δt* (*h*). *A* is the membrane contact area (m^2^). In addition, the separation factor (β) allows to calculate the membrane permeability for the specific component *i*, which is described by Equation (13).

(13) $$\beta_{ii}=\frac{c^{\prime }\left(1-c\right)}{c\left(1-c^{\prime }\right)},$$

where *β_ii_* is the separation factor for component *i* and *c’* and *c* are the aroma compounds concentration in permeate and feed side, respectively. Moreover, where temperature and pressure are used as a driving force in the pervaporation process, the molar flux of ethanol can be given by Equation (14) (Paredes et al., 2024).

(14) $$j_{Et}=\left(\frac{P_{Et}}{l}\right)\;\left(x_{Et}\gamma_{Et}p_{Et}^{f}-Y_{Et}p\right),$$

where $j_{Et}$ is the molar flux of ethanol (mol m^−2^ h^−1^), $\left(\frac{P_{Et}}{l}\right)$ is the permeance for ethanol (mol m^−2^ h^−1^ kPa^−1^), and $x_{Et}$ is the ethanol molar fraction in feed. *𝒴_Et_* is the molar fraction of ethanol in permeate, $\gamma_{Et}$ is the activity coefficient for the ethanol, $p_{Et}^{f}$ is the saturated vapor pressure for the ethanol (kPa), and p is the permeate pressure (kPa).

Takács et al. (2007) studied the effect of temperature on wine dealcoholization using pervaporation at a laboratory scale, highlighting its critical role in producing low‐alcohol and alcohol‐free wines. As temperature increased, membrane efficiency and separation ability declined, leading to a higher permeation rate with less desirable products. According to Catarino et al. (2009), feed temperature had the greatest impact on permeate flux, followed by permeate pressure and feed velocity. Permeate flux increased with temperature and feed velocity but decreased with permeate pressure. Using polydimethylsiloxane (PDMS) hollow fiber membranes, V. García et al. (2008) investigated how different operating parameters, such as feed concentration, temperature, and flow rate, affected the amount of bilberry scent that could be extracted from a model solution of water and ethanol. However, like other evaporative technologies, pervaporation also leads to a loss of endogenous water as reported in Table 2.

Pervaporation is a highly selective membrane technique and has proven effective in separating specific solutes, such as aroma compounds by using a hydrophobic membrane (Castro‐Muñoz et al., 2023) and has been used in conjunction with other technologies to recover these compounds from wine. Catarino and Mendes (2011b) and Olmo et al. (2014) in their studies used pervaporation to extract beer aroma compounds, which were then added to non‐alcoholic beer produced by SCC distillation to enhance its aroma quality. The study by Takács et al. (2007) focused on the implementation of pervaporation as a means of reducing the alcohol content of wine. The study used semi‐sweet Tokaji Hárslevelű wine (vintage 1997) for dealcoholization, starting with 13.11% (v/v) alcohol. Through pervaporation, the alcohol content was reduced to less than 0.5% (v/v), while the extracted ethanol reached 35%–38%. However, around 70% of the wine's aroma compounds were lost in the process, concentrating in the removed alcohol. The authors also estimated the operating costs for pilot‐scale production and highlighted the significant investment required, primarily due to the high cost of pervaporation membranes (Takács et al., 2007). Moreover, using byproducts such as the separated alcohol concentrate can greatly improve profitability, as it serves as a valuable raw material for producing wine distillates or industrial spirits (Castro‐Muñoz, 2019a).

Sun et al. (2020) investigated the production of alcohol‐free wine and grape spirit from Cabernet Sauvignon red wine (12.5% (v/v)) using pervaporation membrane technology, employing a two‐stage membrane system, where the first stage removed ethanol and volatile aroma compounds to obtain alcohol‐free wine (<0.5% (v/v)), while the second stage concentrated the ethanol‐rich permeate to produce grape spirit (>50% (v/v)). The process effectively preserved 65%–70% of the aroma compounds, minimizing thermal degradation compared to traditional distillation, while chemical composition analysis revealed increased total acidity, residual sugars, and SO₂ in the alcohol‐free wine, alongside enrichment of esters and higher alcohols in the grape spirit (Sun et al., 2020), which, in addition to its enhanced sensory attributes, offers economic potential as a valuable raw material for producing wine distillates or industrial spirits, thereby improving overall profitability (Castro‐Muñoz, 2019a). Pervaporation enables significant ethanol reduction; however, its effectiveness in preserving wine's sensory complexity is limited due to substantial aroma losses, as many volatile compounds concentrate in the removed ethanol fraction.

### 2.6 Diafiltration

**FIGURE 7:**
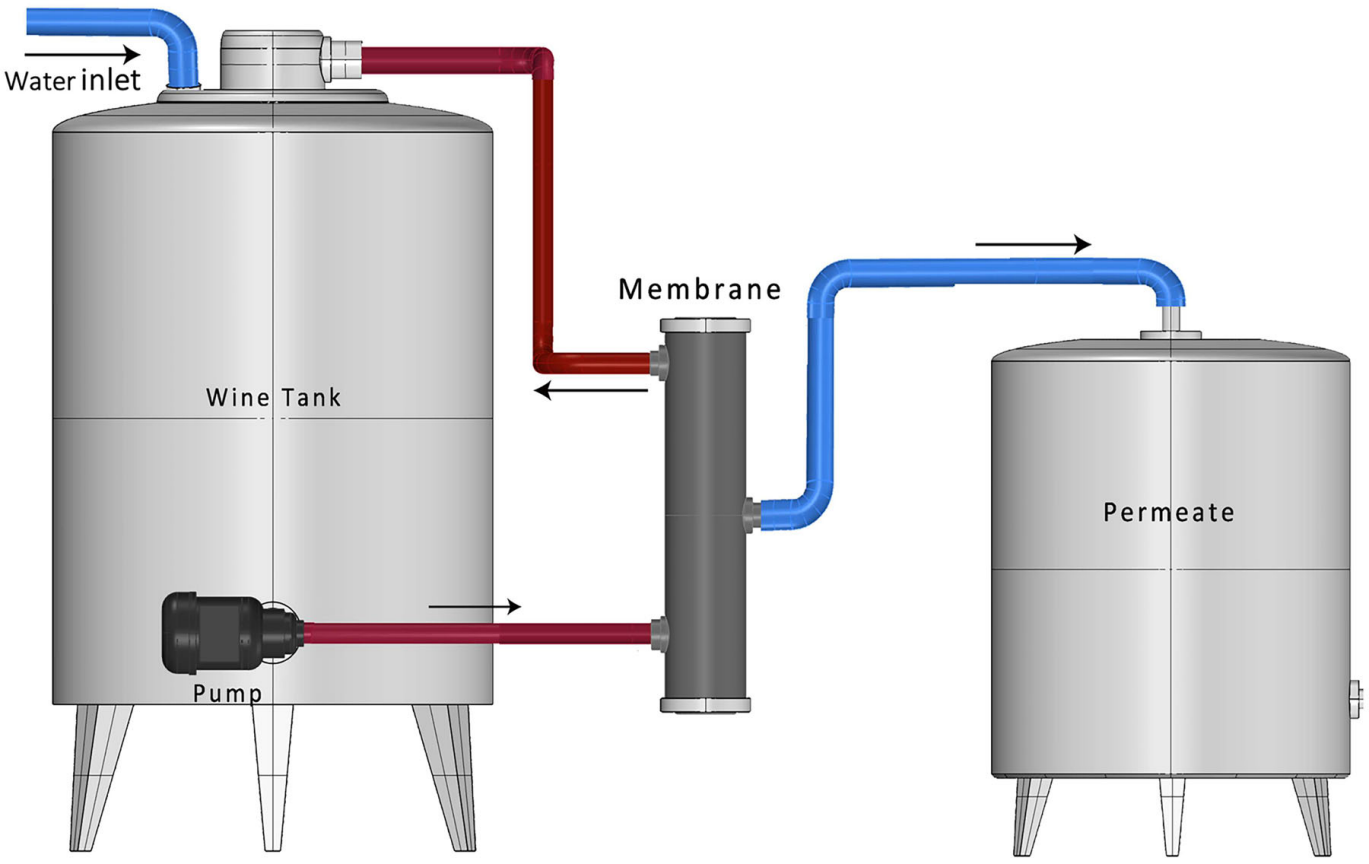
Illustration of the diafiltration technique for dealcoholization, depicting the filtration process where ethanol is extracted through membrane separation, followed by the addition of fresh water to restore balance.

Diafiltration is a membrane‐based technique that works on the principle of filtration and dilution. The general description of the process is illustrated in Figure 7. In the case of wine dealcoholization, wine is passed through the membrane allowing the alcohol to pass through it while retaining the other wine constituents. External water is added to the wine to keep the original volume constant depending on the model of the diafiltration process, which can be continuous (the wine feed volume is kept constant by adding water at a rate equal to permeate) or discontinuous (water is added after the filtration to bring the wine volume to its original) (Castro‐Muñoz, 2020; Takács et al., 2010). The study by Catrino employed a nanofiltration membrane in a diafiltration process, and the experiment was carried out in semi‐continuous mode for wine dealcoholization. The pressure difference across the membrane causes the permeate, mainly consisting of ethanol and water, to pass through it. The volume of the feed tank remained constant with the addition of the deaerated water batch‐wise during the process keeping the concentration of the nonpermeating constituent of wine constant and at the same time decreasing the level of alcohol (Catarino & Mendes, 2011a). Solvent flux and retention coefficient on the nanofiltration membrane can be calculated by Equations (3) and (4) reported above.

Takács et al. (2010) used nanofiltration membranes (NF‐45 and NF‐200) in the diafiltration process. The concentrated wine obtained after the filtration was re‐diluted with water to its original volume. The results indicate that increasing the filtrate flux improves the efficiency of alcohol separation, and the filtration conducted on NF‐200 membranes was found to be more effective due to its lower alcohol retention. Chemical analysis of the dealcoholized wine and permeate indicates 53% of the aroma compound loss. In addition, Ambrosi et al. (2020) used forward osmosis diafiltration to dealcoholized the beer. The process resulted in the permeation of water and alcohol simultaneously through the membrane followed by the addition of water to the retentate thereby reducing the alcohol content of the beer. The obtained dealcoholized beer 0.5% (v/v) had impaired sensory properties affected due to the change in turbidity (increased by 44%), salinity (increased by 70%), color (decreased by 7%), and loss of aroma compounds.

Another study employed diafiltration‐based NF and RO to evaluate their effectiveness in dealcoholizing Mavrud red wine (13.0% (v/v)) while preserving key bioactive compounds. The findings confirmed that the NF (NF99HF) membrane was optimal for moderate dealcoholization, maintaining sensory and bioactive properties, while sequential NF/RO filtration enabled greater ethanol reduction with minimal impact on wine composition (Tsibranska et al., 2025). The application of diafiltration for the dealcoholization of wine has been minimally explored in the literature, with the technique predominantly employed for the separation and purification of polysaccharides and macromolecular fractions in wine (de Bruijn et al., 2011; Resende et al., 2013). Diafiltration is generally a cost‐effective technique in terms of equipment and operational expenses, as it does not require vacuum and heating processes and involves fewer operational steps compared to other dealcoholization methods. While diafiltration can be employed for wine dealcoholization, the method involves the addition of external water to dilute the wine, equivalent to the volume of the permeate removed. However, this practice is strictly prohibited in winemaking regulations, making diafiltration an unsuitable approach for alcohol reduction in wine.

Table 3 summarizes the various dealcoholization techniques, including their specific operating conditions, the types of membranes or instruments used, the types of wine processed, and the initial and final alcohol levels. It also highlights the sensory and chemical changes observed in wine because of alcohol removal, along with corresponding references for each technique. The wine‐drinking experience is influenced by a multitude of factors comprehended in Table 4 that collectively contribute to the enjoyment and satisfaction of the individual (Li & Xia, 2023). Dealcoholization techniques have a significant impact on wine quality, influencing both its volatile and phenolic compositions. Compared to volatile compounds such as esters and higher alcohols, the extent of the changes observed in phenolic compounds is less (Belisario‐Sánchez et al., 2009). Dealcoholization techniques can lead to changes in the perception of astringency, acidity, and overall flavor balance, indicating a complex correlation between alcohol reduction and wine quality (Sam, Ma, Salifu, et al., 2021).

**TABLE 3:** A comprehensive summary of various dealcoholization techniques, detailing their driving forces, membrane types, operating conditions, and equipment used.

Technique	Driving force	Membrane type/dealcoholizing unit	Operating conditions	Wine type	Initial alcohol content	Alcohol content in dealcoholized wine	Reported changes in dealcoholized wines	References
Reverse osmosis	Pressure	Cellulose triacetate/diacetate blend on polyester	P: 16 bar T: 30°C	Red wine	12% (v/v)	7%–8% (v/v)	Loss of aroma compounds Loss of Acid (tartaric acid)	(Catarino & Mendes, 2011a)
		0.5 m^2^ polysulfone spiral membrane	P: 30 bar T: Room temperature	Red wines (Cabernet Sauvignon, Merlot, and Tempranillo varieties)	12.7% (v/v)	Two fractions of 4% and 2% (v/v)	Over 90% retention of glucose main organic acids and glycerol Increased volatile acidity No effect on pH, fructose, and tartaric content Free and total SO_2_ content fall by 25% and 75% for 4% (v/v) and 2% (v/v), respectively No significant difference was found in total anthocyanins and phenolic acid content except in flavanols	(Bogianchini et al., 2011)
		10 spiral wound 4040 RO membranes (nominal MWCO of 220–270 atomic mass units; filtering area 75 m^2^)	P: 3000 kPa T: 45–55°C	Red wines (A: 2014 Barossa Valley Shiraz Cabernet Sauvignon, B: 2015 McLaren Vale Cabernet Sauvignon and C: 2015 Adelaide Hills Shiraz)	A: 14.1% (v/v) B: 17.1% (v/v) C: 14.9% (v/v)	A: 12.5% (v/v) B: 14.5% (v/v) C: 14.2% (v/v)	No significant change in pH, TA, VA, and organic acid A significant change in color for wine A as compared to B and C Loss of free SO_2_ Reduction in volatile compounds	(Pham et al., 2019)
		Spiral‐wound cellular acetate membranes	P: 35–50 bar T: 0°C	Homemade alcoholic wine	5.5%–7.1% (v/v)	0.5% (v/v)	Inconsistent product quality	(Pilipovik & Riverol, 2005)
		Memstar AA MEM‐074, equipped with a microporous polypropylene hollow fiber membrane Celgard X50 in membrane contactor module	—	Red wine (Shiraz)	16.5% (v/v)	10.5% (v/v) 13.5% (v/v)	No changes in pH, TA, malic acid, acetic acid, and glycerol following dealcoholization A significant difference in volatile compounds was observed Concentration of esters and compounds such as ethyl octanoate, ethyl decanoate, ethyl‐2‐methyl butyrate and isoamyl acetate decreased, whereas relative stability found for isobutanol and phenylethanol, concentration of lactones remained stable, and concentration of monoterpenes decreased following dealcoholization	(Longo et al., 2018b)
		Six spiral wound Alfa Laval RO98pHt M20 composite membranes	P: 3.5 MPa, T: 20°C	A: Red wine (Merlot) B: Rose wine (Pinot Noir) C: White wine (Chardonnay)	A: 13.9% (v/v), B: 12.2% (v/v) C: 13.4% (v/v)	Final alcohol content 0.7% (v/v)	Significant loss in concentrations of volatile acidity, free SO_2_, total SO_2_, and pH Increase in color intensity and decrease in hue Significant loss in esters, higher alcohols, organic acids, and terpenes	(Sam, Ma, Liang, et al., 2021)
		—	P: 60–80 bar T: 22–25°C	—	15% (v/v)	13.26% (v/v)	—	(Margallo et al., 2015)
		Oliversep 4 RO kit with eight double membranes	—	Red wine (Cabernet Sauvignon and Listán Prieto)	—	0.5% and 6% (v/v)	—	(R. L. García et al., 2021)
Osmotic distillation	Vapor pressure gradient/concentration gradient	Hydrophobic hollow fiber membrane equips with 1.7 × 5.5, Liqui‐Cel.	Atmospheric pressure and room temperature	Red wine (Montepulciano d'Abruzzo)	13.2% (v/v)	Up to 2.7% (v/v)	No significant changes in color, total anthocyanins, flavonoids, and phenols Reduction in volatile content with increased dealcoholization Higher ester retention in dealcoholized wines compared to alcohols No significant differences (*p* < 0.05) in total volatile acidity, color, total polyphenol content, and organic acids content	(Corona et al., 2019)
		Liqui‐Cel MM‐1 × 5.5 hydrophobic porous polypropylene hollow fiber membranes	Atmospheric pressure and room temperature	Red wine (tempranillo)	14.5% (v/v)	11.5% (v/v)	—	(Esteras‐Saz et al., 2021)
		Polypropylene hollow fiber membrane contactor (Liqui‐Cel Extra‐Flow 2.5 in. × 8 in., 1.4 m^2^, Celgard)	P: 0.4 bar T: 25°C	Red wine (Merlot)	13.35% (v/v)	11.3% (v/v)	Aroma compound losses increase with higher retention time, potentially leading to almost total disappearance Some flavors like 2‐phenylethanol and 2‐phenylethyl acetate maintained their initial concentrations	(Diban et al., 2008)
		1 × 5.5 minimodule (Liqui‐Cel) equipped with microporous hydrophobic polypropylene membrane	T: 30°C	White wine (Falanghina)	12.5% (v/v)	Reduced up to 0.3% (v/v)	No significant changes in TA, pH, organic acids, color, total phenols, and flavanols Volatile compounds (higher alcohols, esters and acids) decreased with increasing alcohol removal with an overall loss of 96% in totally dealcoholized wine (0.3% (v/v))	(Liguori et al., 2019)
		1 × 5.5 minimodule (Liqui‐Cel) Hollow fiber polypropylene membrane	T: 20°C	Red wine (Aglianico)	13% (v/v)	Final alcohol content reduced to 0.19% (v/v)	No significant changes in total phenols, flavonols, tartaric esters, and organic acids (*p* < 0.05) Significant decrease in the volatile fraction with increasing dealcoholization level Increase in color intensity and tonality with deeper alcohol reduction (higher than − 6.5% (v/v))	(Liguori et al., 2013b)
		Liqui‐Cel (MM‐1 × 5.5) from 3 M: hydrophobic porous polypropylene hollow fibers, PP2 from Zena: higher pore size PP‐based hollow fibers, PVDF from Polymem: superhydrophobic PVDF hollow fibers	P: 1 atm T: 11–15°C	Red wines (Tempranillo)	14.8% (v/v) 14.2% (v/v) 14.1% (v/v)	11.8% (v/v) 11.2% (v/v) 11.2% (v/v)	Aroma loss increased in the order: esters > alcohols > acids Lowering working temperature from 15 to 11°C reduced esters loss without affecting ethanol flux. Initial wine aroma concentrations did not influence OD performance	(Esteras‐Saz et al., 2024)
		Polypropylene hollow fiber membrane contactor (Liqui‐Cel 4 × 25 Extra Flow) provided with a membrane area of 20 m^2^	Constant atmospheric pressure and room temperature (15)	Red wine (Garnacha and Tempranillo) White wine (Xarelo)	10.1 % (v/v), 9.3% (v/v), 9% (v/v)	Reduction of −2 % (v/v) from original alcohol content	Aroma compound losses maintained at 25%–37% Potential reduction of aroma losses to ∼20% Predicted aroma losses below 20% under optimized industrial conditions	(Diban et al., 2013)
		Rectangular 0.5 × 1 Micromodule (Liqui‐Cel) equipped with polypropylene hollow fiber membrane	T: 20	Red wine (Aglianico)	12.5% (v/v)	10.65% (v/v)	No significant differences in the main parameters (TA, VA, polyphenols, and organic acids) were reported. Color and tonality values remained unchanged after dealcoholization	(Liguori et al., 2013a)
Vacuum distillation	Reduced pressure	Vacuum distiller by REDA S.p.A. (Isola Vicentina)	T: 15°C	Rosè (Langhe) Red wines (Verduno, Pelaverga, and Barbera)	13.2% (v/v) 15.2% (v/v) 14.6% (v/v)	5% (v/v)	Dealcoholized wine had higher contents of total anthocyanins, total flavonoids, and organic acids; total extract and ashes; and lower pH Increased color intensity and decreased hue Major loss of volatile compounds was from higher alcohols (isoamyl alcohol (61.8%–68.7%), isobutanol (59%–69%), cis‐3‐hexenol (51.7%–69%) and n‐hexanol (39%–69%), followed by esters (ethyl hexanoate, ethyl octanoate and ethyl decanoate), acetate (isoamyl and phenylethyl acetate) and acids (isovaleric acid) Wine fractions dealcoholized at 5% (v/v) maintained wine‐like composition	(Motta et al., 2017)
		Vacuum evaporator (Reda Concentrator mod CM100)	P: 30 mbar, T: 18–25°C	White wine (Chardonnay)	10.79% (v/v)	0.1% (v/v)	The main volatile compounds that underwent the highest losses were the acetates of higher alcohols (especially isoamyl acetate) and the ethyl esters of medium‐chain fatty acids (ethyl hexanoate and ethyl octanoate)	(Petrozziello et al., 2019)
		Industrial System operated in vacuum (unspecified)	Vacuum: 50–60 mmHg, T: 25°C	White wine	10.6% (v/v)	0.3% (v/v)	The final product lacked in most of the volatile compounds Some volatile compounds that could be detected were found in lower concentrations than in the original wine Esters like diethyl succinate, 2‐phenylethyl acetate, and 2 propenyl benzene acetate almost maintained their initial concentration in the dealcoholized wine	(Gómez‐Plaza et al., 1999)
		Laboratory‐scale distillation plant, RE‐6000A rotary evaporator (Shanghai Yarong Biochemical Instrument Factory)	Vacuum pressure: 0.08 MPa, T: 35°C	Red wine (Merlot), Rosé wine (Pinot noir), White wine (Chardonnay)	13.9% (v/v), 12.2% (v/v), 13.4% (v/v)	0.7% (v/v)	Higher sugar concentration, increased acidity and significant loss of VA, free SO_2_, total SO_2_, and pH in the dealcoholized wine fractions Increased in color intensity and decreased in hue Significant loss of volatile compounds such as esters (loss of 96%, 98%, and 96% in white, rosé, and red wines, respectively), higher alcohols (about 95%, 85%, and 94% losses in white, rosé, and red wines, respectively), organic acid (lost by 85% and 91% in white and red wines, respectively), terpenes and C_13_‐norisoprenoids (lost by 92%–100%) and carbonyl compounds	(Sam, Ma, Liang, et al., 2021)
Spinning cone column	Centrifugal force	SCC distillation pilot plant manufactured by Conetech (production capacity is 1000 L/8 h, column diameter and height are 0.33 and 2 m, respectively)	Vacuum: <32 mmHg, T: 26–30°C	Red wines (Petit Verdot, Garnacha, Syrah, Monastrell, Monastrell Condomina, Tempranillo, Crianza, Joven, Merlot, and Cabernet Sauvignon), Rosé wines (Caber Sauvignon, and Bobal), White wines (Macabeo, Malvar, Moscatel Romano, and Macabeo and Airén)	11.95%–14% (v/v)	0.1% (v/v) (without aroma fraction), 0.5% (v/v) (with added aroma fraction)	All the red and rosé dealcoholized wine exhibited the same or increased (*p* ≤ 0.05) in the content of anthocyanins Resveratrol content was found to be either same or higher (this was because of ethanol removal) Loss of free SO_2_ is confirmed by the percentage of remaining DPPH^•^ or decrease in antioxidant activity	(Belisario‐Sánchez et al., 2009)
		Unspecified	—	Red Wine (Listán Prieto)	—	0.5% and 6% (v/v)	—	(R. L. García et al., 2021)
		SCC distillation pilot plant (Conetech), the column has 0.33 m in diameter and 2 m in height	Vacuum: 32 mmHg, T: 26–30°C	Red wine (Tempranillo), Rose wine (Cabernet Sauvignon), White wine (Chardonnay)	14% (v/v)	Final alcohol content 0.5% (v/v)	Maximum aromatic (volatile compounds) extraction	(Belisario‐Sánchez et al., 2012)
Pervaporation	Vapor pressure difference	Pilot‐scale pervaporation equipment equipped with two‐stage membrane system; Spiral wound PV membrane (JS‐WSM‐8040) made of two layers polyacrylonitrile (PAN) and polydimethylsiloxane (PDMS)	P: 5 kPa T: 45°C Condensation temperature: −15°C	Red wine (Cabernet Sauvignon)	12.5% (v/v)	<0.5% (v/v)	Two‐stage process to balance aroma compounds in dealcoholized wine Higher temperatures can cause a steamed taste, affecting the quality of alcohol‐free wine Higher alcohols and esters increased in the permeate compared to the original wine Aroma constituents in the permeate were 70% of the total aroma content of raw wine TA, dry extract, and residual sugar increased in the retentate Ethanol and aroma compounds are vaporized and condensed to create distilled liquor	(Sun et al., 2020)
		Laboratory scale pervaporation equipment constructed by Corvinus University of Budapest and produced by Hidrofilt Ltd. The membrane module was equipped with the PERVAP. Sulzer 1060 type organophilic flat composite membrane made of PDMS	P: Temperature range used: 40–70°C	White wine (semi‐sweet Tokaji Hárslevelű)	13.11% (v/v)	0.05% (v/v)	Permeate production gets faster, but less desired product is gained by separation at higher temperatures 70% loss of aroma compounds in dealcoholized wine Lower pervaporation temperatures are preferred to prevent significant aroma loss	(Takács et al., 2007)
		Composite polyoctylmethylsiloxane/polyetherimide (POMS/PEI) membrane	P: 1.0 mbar, T: 12°C	Red wine	12% (v/v)	0.3% (v/v) (aroma extract)	Adding extracted aromas boosted volatile compound levels in dealcoholized wine Concentration of esters increased in corrected dealcoholized wine	(Catarino & Mendes, 2011a)
		CELFA Laboratory P‐28 (CM‐Celfa Membrantrenntechnik AG) equipped with PDMS membrane (Pervatech BV Netherland)	Permeate pressure: 0.013 bar, Three temperature conditions: 35, 40, and 45°C	Red wine (Malbec)	14.8% (v/v)	10.5% (v/v) Blend 13.5% (v/v)	Minute difference in pH but no effect on pH at different operating temperatures Increase in TA and VA Reduction in anthocyanins and tannins Reduction of all volatile organic compounds	(Paredes et al., 2024)
Diafiltration	Transmembrane pressure difference	Polyamide	25°C 5–20 bar	A: Red Wine (Egri Bikavér), B: White wine (Tokaji Hárslevelű)	A: 11.25% (v/v), B: 13.11% (v/v)	—	The obtained dealcoholized wine retained 47% of aroma compounds Four of the acids (hexanoic, octanoic, hexadienoic, and decanoic acid) identified in the wine were lost in the end product	(Takács et al., 2010)

*Note*: The table highlights the types of wines processed, initial and final alcohol content, and reported changes in wine characteristics after dealcoholization. The data offer insights into how different methods impact aroma compounds, acidity, color, and volatile composition, emphasizing the challenges and trade‐offs in reducing alcohol content while maintaining wine quality.

Abbreviations: P, pressure; PDMS, polydimethylsiloxane; T, temperature; TA, total acidity; VA, volatile acidity.

**TABLE 4:** Key sensory parameters that influence wine acceptability among consumers.

Sensory parameters	Factors involving the acceptability of wine
Appearance	Wine's visual characteristics, such as color, clarity, and intensity Preferences for specific color hues or intensity levels
Aroma	Desirable aromas can include fruity, floral, herbal, or spicy notes Aroma profile intensity, complexity, and distinctiveness Presence of any off‐putting or faulty aromas result in unacceptability
Taste	Taste profile of wine, including its sweetness, acidity, bitterness, tannins (in red wines), and overall balance Perception of sweetness and acidity (affects overall taste balance) Individual taste preference
Mouthfeel	Mouthfeel of wine refers to its tactile sensations in the mouth, such as body, texture, and astringency Alcohol content, residual sugar, and tannins influence the wine's mouthfeel
Finish	Sensations and flavors that linger after swallowing A balanced and complex finish, which is associated with higher quality and sensory acceptability
Overall complexity and harmony	Wine with range of flavors and aromas, with a well‐integrated and harmonious profile Complexity and balance contribute to the overall quality perception

*Note*: The table details how appearance, aroma, taste, mouthfeel, finish, and overall complexity contribute to the perception of wine quality.

## 3 DEALCOHOLIZED PRODUCTS MARKET

**FIGURE 8:**
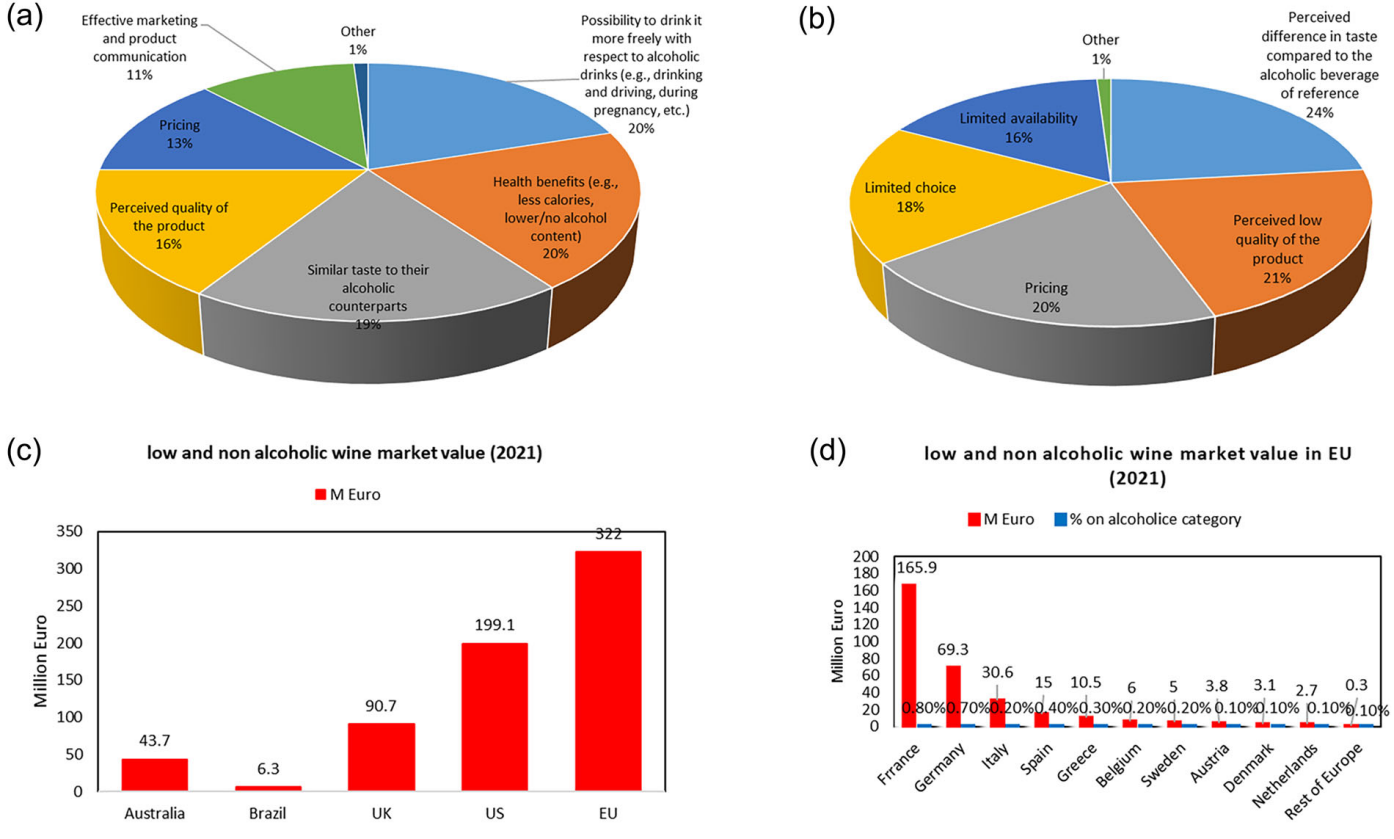
(a) European Union (EU) consumers’ main reasons encouraging to try low‐/no‐alcohol beverages, (b) EU consumers’ main reasons discouraging to try low‐/no‐alcohol beverages, (c) estimated market size of low‐ and no‐alcohol wine in major wine‐producing and wine‐consuming region, (d) main EU markets of low‐ and no‐alcohol wine in different EU countries (Adapted from the data reported in final report published by the European commission on “study on low/no alcohol beverages”; Gentile et al., 2023).

In nations adhering to Islamic law, the consumption of alcohol is strictly prohibited. As a substitute for traditional beer and other non‐alcoholic beverages, non‐alcoholic beers (NABs) have emerged as a viable option. These NABs contain less than 0.5% (v/v) alcohol and offer similar bioactive components as their alcoholic counterparts (Muller et al., 2019). Similarly, dealcoholized wine can have a potential market in such countries as an alternative to traditional alcoholic beverages. In addition, dealcoholized products may lower health risks by reducing alcohol intake. This strategy can help to decrease the general population's consumption of alcohol and switch from stronger to weaker alcoholic beverages like replacing them with dealcoholized and partially dealcoholized wine. Besides, regular consumers can also replace conventional alcoholic drinks with low‐alcohol wines as an alternative to reducing their average alcohol intake (Aepli, 2014; Rehm et al., 2016, 2023). Figure 8a,b reports some imperative reasons from survey results and interviews that currently encourage and discourage customers’ interest in dealcoholized and non‐alcoholic beverages, respectively. The top three reasons for the acceptance of these products were the possibility to drink freely, health benefits, and similar taste profile, while the major reasons that deterred were different taste, low quality, and high price (Gentile et al., 2023).

Bucher et al. (2020) examined the impact of low‐alcohol wine on consumer perception and behavior. Their findings indicated that consumers perceived low‐alcohol wine as comparable to standard wine. Recent improvements in sensory quality and increasing awareness of health‐related benefits may have contributed to a higher acceptance of wines with reduced alcohol levels, which could explain the positive evaluation. However, despite the growing market for low‐alcohol wines, consumers might not be ready to pay the same or a premium for dealcoholized products (Bucher et al., 2020). Several countries have imposed taxes on alcoholic beverages with respect to their alcohol content level (Roche et al., 2023). Likewise, the United Kingdom's current system of taxing alcoholic beverages is going to be changed to charge all types of alcoholic drinks according to their alcohol level, which will help in reducing the dealcoholized wine price in the UK market.

In the 2020 Social Market Foundation report, 27% of UK adults had consumed dealcoholized products in the past year, with 41% saying it had led them to reduce alcohol intake, 44% saying it was unchanged, and 6% saying it had increased (Pardoe, 2020). Another survey report on UK adults with heavy drinking habits found that over 53% of drinkers in the survey considered these products essential or very important to their efforts to cut back. Most of the people in the survey (98.5%) were trying to cut back on alcohol levels (Piper & Leyshon, 2023). Dealcoholized wines are one of the fastest‐growing beverage categories globally. Alcoholic beverages that were dealcoholized and have low alcohol by volume dominate this novel product category. According to International Wine and Spirit Records (IWSR), 2022 global sales hit US$22 billion for all types of dealcoholized beverages. The IWSR expects 7% annual sales growth for the next 5 years, with 90% of the beverage industry growing. With new technological developments in wine industries, dealcoholized wine quality has improved in recent years. Although the current market for dealcoholized wine is small compared to dealcoholized beer, the market share is growing rapidly in several countries with each passing year (Mueller Loose, 2023).

The report published by the European Commission (study on low‐/no‐alcohol beverages market 2023) comprehensively covered all the aspects of low‐/no‐alcohol beverages from legislation, market share, market potential, consumer surveys on liking, and disliking to future expectations. Low‐ and no‐alcohol wine is worth ∼€322 million and consisted of 42 million liters in 2021, representing a volume and value share of 0.4% of the total EU wine market. The market value for low and no‐alcohol wines in five major wine‐consuming regions is reported in Figure 8c, where the European Union is the biggest market followed by United States €199.1 M, the United Kingdom €90.7 M, Australia €43.7 M, and Brazil €6 .3 M. Within Europe, the biggest market for dealcoholized, partially dealcoholized, and no‐alcohol wine is France encompassing €165.9 M worth followed by Germany €69.3 M, Italy €30.6 M, and Spain €15 M (Figure 8d). According to the report, recent EU regulations to produce dealcoholized and partially dealcoholized wine will boom an anticipated 25% increase in the demand for these products in the following years (Gentile et al., 2023). As per wine traders and merchants, approximately one‐third of the wine business perceives dealcoholized or low‐alcohol wines as trendy in the following year. In general, partially dealcoholized wines are seen by merchants as having more potential than no‐alcohol wines (Table 5), whereas there are substantial regional variations among nations (Mueller Loose, 2023).

**TABLE 5:** Top 10 markets for partially dealcoholized/low‐alcohol and dealcoholized/no‐alcohol wines, based on expectations from wine traders and merchants for the upcoming year.

Ranking	Country	Partially dealcoholized/low‐alcohol wine (%)	Dealcoholized/no‐alcohol wine (%)
1	UK	67	53
2	Netherlands	56	43
3	Finland	51	36
4	Germany	43	34
5	Norway	36	33
6	Belgium	34	24
7	Denmark	31	22
8	Spain	31	21
9	USA	28	20
10	Switzerland	28	19

*Note*: The rankings highlight countries where demand is projected to be highest, with the United Kingdom, Netherlands, and Finland leading the market. The percentages represent the anticipated market interest in each category, reflecting the growing global trend toward low‐ and no‐alcohol wine consumption (Mueller Loose, 2023) (Adapted from the special Report on ProWein Business Survey 2022).

Moreover, the expected rise in sales of dealcoholized wine can be increased by raising consumer awareness about high alcohol consumption and most importantly by the improvement of the sensory characteristics of dealcoholized wine. It is imperative to acknowledge that the methods employed to enhance sensory attributes depend on the original composition of the wine, the technique employed for dealcoholizing, and the intended flavor profile. Winemakers can employ a combination of these techniques and methodologies and undertake sensory assessments to attain optimal products. Moreover, the integration of consumer feedback holds significant value in the process of improving the sensory attributes to align with the discerning tastes and preferences of the market.

## 4 DRAWBACKS OF WINE DEALCOHOLIZATION

Dealcoholized wine, although appealing to individuals seeking to abstain from alcohol, may also possess some drawbacks regarding its stability and sensory and chemical profile of dealcoholized wine.

### 4.1 Stability of dealcoholized wines

Alcohol serves as an inherent preservative in wine, and eliminating alcohol diminishes the preservative impact, which could increase the vulnerability of dealcoholized wine to microbiological, oxidative, and chemical instability.

#### 4.1.1 Microbial stability

Regardless of the technology employed to produce wines with reduced alcohol content, manufacturers face a considerable challenge in maintaining product stability, extending shelf life, and ensuring timely market distribution. While microbes play a crucial role in fermentation, certain yeasts and bacteria can proliferate under favorable conditions, leading to wine spoilage and a decline in quality and commercial value (Bartowsky, 2009; Branco et al., 2021). Wine spoilage can lead to haze development, elevated levels of acetic acid and volatile acidity, higher viscosity, and the development of off odors caused by volatile molecules, for example, volatile phenols, ethyl acetate (Bartowsky, 2009). Acetic acid and lactic acid bacteria are commonly considered to be causing wine spoilage, but some lactic acid bacteria have found useful applications in winemaking (e.g., *Lactobacillus plantarum* and *Oenococcus oeni* in malolactic fermentation) (Izquierdo‐Cañas et al., 2020; Sabel et al., 2017; Virdis et al., 2021). The susceptibility of wine to spoilage is influenced by various factors such as ethanol content (the second major constituent after water in wine and acts as a preservative in wine), residual sugar concentration, pH, acidity levels (e.g., malic acid), oxygen levels, types of yeast/bacteria present, stabilization treatments, and the amount and type of chemical preservatives used (Bartowsky, 2009; Du Toit & Pretorius, 2000).

Sulfur dioxide and sodium metabisulfite are utilized in winemaking and storage for their antibacterial and antioxidant qualities (Sánchez‐Rubio et al., 2017). As many subjects are intolerant or sensitive to sulfites even at modest concentrations, their use is strictly regulated in food products (Vally et al., 2009). Therefore, weak acids like benzoic, sorbic, and dimethyl dicarbonate could be suitable substitutes for sulfites to preserve dealcoholized wines (Sánchez‐Rubio et al., 2017). However, in dealcoholized wine where eliminating ethanol from wine renders it particularly vulnerable to microbial proliferation, adding preservatives in wine in legal quantities might not be enough, therefore necessitating the use of more tightly regulated packaging conditions to maintain the integrity of the product and might require proper pasteurization and use of preservatives combined to prolong the shelf life of the dealcoholized wine (Salvador et al., 1992; Taboad, 2013). Salvador et al. (1992) applied flash pasteurization on dealcoholized wine to increase the shelf life and compared it with chemically preserved and aseptic filtered wines. Wine sensory analysis results exhibited that wine preserved by flash pasteurization or aseptic filtration was superior to those preserved using chemicals. Pasteurization can be useful to preserve dealcoholized wine, but scientific studies on the quality of dealcoholized wine after pasteurization are quite scarce, thus requiring further attention of the scientific community. Moreover, various cutting‐edge technologies, including microwave technology, pulsed electric field technology, ultraviolet irradiation, high pressure, and ultrasound, have been explored for their efficacy in eliminating undesirable microorganisms in wine (Pinto et al., 2020). Physical methods may be more suitable than chemical additives for reducing the use of chemicals in wine.

#### 4.1.2 Oxidative stability

Dealcoholized wines are sensitive to oxidative degradation. Controlled oxidation in red wine can help preserve and enhance color while reducing astringency, thereby improving its overall sensory qualities. Oxidation can significantly reduce anthocyanin content and induce browning, both of which are undesirable in wines. The degradation of anthocyanins is known to accelerate markedly in the presence of oxygen (Escribano‐Bailón et al., 2019). O‐Diphenols play a crucial role in scavenging oxygen and protecting wine flavors from oxidative degradation. Acetaldehyde is produced indirectly through the interaction between phenols and oxygen, promoting flavanol polymerization with oxidation‐sensitive anthocyanins, ultimately forming oxidation‐resistant anthocyanin–tannin complexes (Jackson, 2016). An oxygen reduction product, the hydroxyl radical, can oxidize nearly any organic compound in wine, including ethanol, converting it into acetaldehyde or other organic acids (Waterhouse & Laurie, 2006). Due to its lack of selectivity, the hydroxyl radical reacts with the first available species, with its impact depending on their concentration (Danilewicz, 2007). Consequently, the partial or complete removal of alcohol may facilitate the oxidization of phenols, organic acids, and other volatile compounds leading to the formation of off‐flavors and brown pigments.

Sulfur dioxide (SO_2_) is not only used to prevent microbial spoilage but is also a crucial and commonly used chemical for preventing wine oxidation (Sánchez‐Rubio et al., 2017). SO_2_ can also engage in addition reactions with carbonyl compounds to generate non‐volatile bisulfite adducts, which prevents undesirable sensory qualities. In addition, grapes contain ascorbic acid by nature; however, it is consumed rapidly following crushing, owing to its effective oxygen‐scavenging properties; therefore, it is also added particularly to white wine to prevent oxidation reactions (Oliveira et al., 2011). Dealcoholized wines offer a favorable supply of polyphenols with no‐alcohol intake, and one of the studies on the stability of antioxidants after 30 days of storage on commercially dealcoholized wines has seen a considerable drop in polyphenolic content and antioxidant activity in bottle storage (Bogianchini et al., 2011). Therefore, it is advisable to establish and clearly indicate a minimum recommended consumption period on the label. This would help ensure that consumers enjoy the product while it retains its intended chemical and sensory properties, preventing potential quality deterioration over time.

#### 4.1.3 Color stability

Color is a crucial characteristic of wine. Besides water and alcohol, the remaining fraction of wine is composed of distinct colors, flavors, aromas, and residual sugar. Anthocyanins are the primary coloring molecules in red wine, whereas flavanols are responsible for the color in white wine (Fairchild, 2018). The device‐independent color space CIELAB is frequently employed in the analysis of wine color. Bogianchini et al. (2011) reported the effect of RO on the color parameters of red wines. Besides other ecological parameters, decreasing the alcohol concentration resulted in lower CIELAB values. A reduction in the red/green value (a*) resulted in an increased shade of green, while a reduction in the blue/yellow value (b*) caused a greater shade of blue. All these color parameter variations can be readily attributed to the impact of ethanol on copigmentation processes (Bogianchini et al., 2011) which has been explained by Boulton (2001) in his critical review.

The variation in color intensity differs between wines and depends on the dealcoholization technique employed, with several studies documenting changes in wine color following dealcoholization. For instance, OD applied to Aglianico (Liguori et al., 2013b), RO to Merlot (Sam et al., 2023), Lange, Pelaverga, and Barbera wines to membrane‐contractor and VD (Motta et al., 2017), a combined treatment of RO‐OD to Montepulciano d'Abruzzo (Russo et al., 2019), and VD to Merlot and Pinot Noir (Sam, Ma, Liang, et al., 2021) all showed an increase in color intensity, which can be attributed to the concentration effect caused by alcohol removal (Sam, Ma, Salifu, et al., 2021) and the oxidation of wine pigments and compounds. The loss of SO₂ may lead to reduced protection against color degradation (Liguori et al., 2013b). Similarly, as reported by Gambuti et al. (2011), the chromatic properties of wines were not decreased with the loss of monomeric anthocyanins in Merlot (loss of 57%), Piedrosso (loss of 49%) and Aglianico (loss of 49%) during dealcoholization by membrane contactor, likely due to the dominance of copigments and the potential formation of more colored pigments in the dealcoholization process due to oxidation and SO_2_ loss as mentioned earlier.

### 4.2 Potential flavor imbalance

The change in phenolic and volatile organic compounds influences the overall chemical and sensory profile of dealcoholized wine. Phenolic compounds are crucial in shaping the bitterness and astringency of wine. They also play an essential role in preserving wine and form the basis for its potential to age over time (Allegro et al., 2021; Merkytė et al., 2020). Dealcoholization processes typically have minimal impact on the phenolic composition of wine (Belisario‐Sánchez et al., 2009); in addition, dealcoholization by SCC has also reported an increase in phenolic compound concentrations (Belisario‐Sánchez et al., 2009; Bogianchini et al., 2011; Corona et al., 2019; Motta et al., 2017), which is attributed to total volume loss resulting from the removal of alcohol. The reduction of wine volatile organic compounds during dealcoholization is inevitable and predominantly influenced by several factors including operating conditions, concentration gradient across the membrane, and physical and chemical properties of volatile compounds (water solubility, hydrophobicity, membrane affinity, and pore size) alongside their interaction with the wine matrix and alcohol level (Esteras‐Saz et al., 2023; Esteras‐Saz et al., 2021; Longo et al., 2017). RO membranes, with their fine pore size and low molecular weight cut‐off (MWCO), are highly effective at retaining small molecular weight compounds, but this can also lead to higher energy consumption and increased membrane fouling (Labanda et al., 2009).

The rejection and retention of a compound are influenced by its volatility along with its polarity and the hydrophobicity of the membrane (Castro‐Muñoz, 2019b). It is observed that polyamide membranes exhibit higher retention of volatile compounds compared to cellulose acetate membranes. Polar membranes tend to attract polar organic compounds to their surface, resulting in higher permeability. Hydrophobic membranes demonstrated greater rejection of highly polar organic compounds: hexanol, which has a hydrophobic nature, interacts with the hydrophobic regions of the membrane, which increases its permeability, particularly at higher temperatures (López et al., 2002). Diban et al. (2008) used a polypropylene (PP) hollow fiber membrane to study mass transfer kinetics during the dealcoholization of Merlot wine and a model solution. Significant losses of volatile compounds, particularly ethyl octanoate (57.5%–98.1%), were observed due to its high hydrophobicity, which increased its affinity for the membrane and its volatility, leading to evaporation and diffusion into the stripping water.

Polyphenols, a key component of red wine's non‐volatile matrix, can form non‐covalent interactions with aroma compounds through π–π stacking, which is stabilized by hydrogen bonding (Jung et al., 2000). This interaction may account for the stability of 2‐phenylethanol levels observed during a 50% reduction of ethanol concentration in Aglianico wine (13% (v/v)) achieved through a membrane contactor (Liguori et al., 2013b). Conversely, 2‐phenylethanol level was found to be decreased significantly at a higher level of alcohol reduction, likely due to weaker π–π stacking at lower alcohol concentrations. In addition, the aggregation of tannins at lower alcohol concentrations in wine may also decrease the bonding sites for aroma compounds (Poncet‐Legrand et al., 2003). Therefore, alterations in mouthfeel and diminished fragrances observed in low‐alcohol wines can also be attributed to chemical modifications in the structure of volatile compounds and the decreased volatility of esters and higher alcohols resulting from the absence of ethanol (Longo et al., 2018a; Osorio Alises et al., 2023). In addition, the alteration of aroma compounds during thermal distillation has resulted in the advancement of distillation methods that operate at low temperatures (Diban et al., 2013).

### 4.3 Cost implication

The use of dealcoholization techniques in winemaking will potentially cause an increase in production costs which will vary greatly depending upon the technique applied, which is crucial for winemakers to consider when deciding the production approach (Schmitt & Christmann, 2022). Four major factors influence the cost: initial investment, operational expenses, volume loss, and the need for trained labor to operate the process. The initial investment required for acquiring the equipment represents a substantial upfront cost, which can be challenging for smaller wineries to manage effectively. In addition, those wineries could rely on service providers instead of acquiring their equipment for small‐scale production. Operational costs represent another significant expense in the dealcoholization process, with variations across methods due to differing energy requirements and processing times, which directly influence overall production costs. For membrane‐based techniques, additional costs arise from maintenance, membrane cleaning, and periodic replacement, with membrane costs varying significantly depending on the type used. Furthermore, reverse osmosis requires high‐pressure filtration, and the permeate undergoes distillation to recover water, which is reintroduced into the wine. This additional step increases energy consumption, thereby elevating production costs (Paredes et al., 2023). Similarly, techniques such as vacuum distillation (Petrozziello et al., 2019) and spinning cone column (Belisario‐Sánchez et al., 2012) involve heat treatment under reduced pressure, and the production of steam in the case of SCC, to remove alcohol, which demands substantial energy input, further contributing to high operational costs.

Volume loss during dealcoholization is an inevitable cost that must be considered in production planning. Depending on the technique employed and the operational parameters, wineries generally experience a volume reduction depending on technique and level of dealcoholization. As reported by Kumar et al. (2025), the total volume loss to obtain dealcoholized wine of 0.5% (v/v) resulted in 20.7%. This reduction directly impacts overall yield and profitability, making it a critical factor in cost assessments. One potential strategy to offset this loss is recovering the aromatic alcohol fraction, which can be utilized as an aromatic wine spirit especially in VD, SCC, and pervaporation thereby mitigating some of the financial impact. The operational complexity of dealcoholization needs the use of professional and technically qualified personnel to reduce the danger of unexpected losses. This supplementary requirement increases operational expenses, especially for methods like RO, VD, and SCC, where precise control and expertise are essential for effective processing and upholding product quality. Due to the inclusion of these costs on the end product, consumer acceptance could be impacted if dealcoholized wines are priced higher than regular wines (Schmitt & Christmann, 2022; UK, 2023).

## 5 PERSPECTIVES FOR DEALCOHOLIZED WINE

There is no question that wine and alcohol have captured people's attention for recreational, ceremonial, social, and communal gatherings (Phillips, 2014). Dealcoholized wines are becoming more popular, and winemaking industries are responding by using membrane‐based and thermal distillation techniques. These techniques are designed to produce wine with reduced alcohol while keeping its flavor and aroma such as regular wine. Moreover, dealcoholized wine demand has been increasing steadily over the past few years (Mueller Loose, 2023) due to numerous factors, including health‐oriented lifestyles, changing consumer preferences, and a rise in the need for low‐alcohol substitutes (Waehning & Wells, 2024). It presents various perspectives and opportunities within the beverage industry. Dealcoholized wine products are a good alternative for consumers who want to enjoy the sense of flavor and taste without consuming alcohol including individuals who aim to limit their alcohol consumption, expectant mothers, drivers, and those who stick to religious or cultural beliefs that prohibit alcohol consumption (Karunarathna et al., 2024).

The technological dealcoholization process is complex for small‐ and medium‐sized manufacturers to handle on their own, which contributes to the relatively low number of producers. Only a few major, specialized manufacturers provide the systems needed for this operation (Mueller Loose, 2023). The wine industry has substantially evolved in recent years due to advancements in dealcoholization processes. Among the most widely adopted techniques, RO and SCC are the most utilized in the production process (Gil et al., 2013; Puglisi et al., 2022). Membrane‐based dealcoholization techniques offer several advantages over conventional thermal distillation methods, including lower processing temperatures, enhanced membrane selectivity, and reduced environmental impact due to lower energy consumption (Castro‐Muñoz, 2020; Oro et al., 2025). Nonetheless, significant challenges persist despite these advancements. Membrane fouling, the loss of volatile compounds at a high level of alcohol reduction, and the necessity for optimized membrane materials are significant challenges. Future research should focus on enhancing membrane performance, elucidating molecular interactions, and improving membrane selectivity to better maintain wine quality during dealcoholization.

Thermal distillation methods, including VD and SCC, can accomplish complete dealcoholization more swiftly than membrane‐based techniques. Nonetheless, these techniques encounter considerable disadvantages, such as increased loss of volatile aroma compounds and elevated energy consumption, which restrict their extensive utilization. To reduce the loss of aromatic compounds, an alternative method entails extracting the initial aromatic fraction and reintegrating it into the dealcoholized wine (Belisario‐Sánchez et al., 2012; Huerta‐Pérez & Pérez‐Correa, 2018). Researchers are progressively investigating the application of hybrid methodologies to improve the chemical composition of dealcoholized wine, as achieving complete alcohol removal without affecting the wine quality is completely difficult. Pervaporation techniques using hydrophobic membranes, when combined with distillation or other membrane‐based processes, could offer an effective solution for extracting the aromatic fraction from wine and reintegrating it into dealcoholized wine. This combination has the potential to produce high‐quality, dealcoholized wine while preserving desirable sensory characteristics (Castro‐Muñoz, 2019a). Such integrated methods could show considerable potential in enhancing flavor retention, thus responding to industry standards and consumer preferences. A major obstacle to their wider adoption, however, is the higher operational costs linked with implementing such hybrid strategies (Russo et al., 2019).

Looking ahead, these technologies are poised for further innovation to overcome existing limitations. Research on optimizing parameters, integrating advanced membrane materials, and improving energy efficiency will be pivotal. Additionally, as sustainability becomes a priority, future advancements may focus on reducing water and energy consumption during the dealcoholization process. The ability to tailor alcohol reduction levels to specific consumer preferences without compromising wine quality will likely drive wider adoption, cementing dealcoholized wine as a significant category in the global wine market.

These innovative wines offer versatility, making them suitable for a variety of occasions, including social gatherings, corporate events, formal ceremonies, or celebrations, where the availability of both alcoholic and non‐alcoholic options is essential to accommodate diverse consumer preferences. Additionally, these products offer a promising opportunity to enhance food pairing experiences. When consumed alongside meals, they can complement flavors and contribute to overall taste harmony, such as traditional wine pairings. Food and beverage establishments including restaurants, bars, and wine‐focused venues can expand their beverage selections by incorporating dealcoholized wine options that complement particular dishes or cater to specific dietary requirements. The increasing fascination with dealcoholized wine has stimulated inventive approaches in manufacturing methodologies and taste characteristics. Ongoing efforts by winemakers involve the exploration of novel techniques for the extraction of alcohol from wine, while simultaneously retaining its favorable attributes such as olfactory perception, gustatory sensation, and tactile experience. The diversification of dealcoholized wine styles, including red, white, sparkling, and rosé, has been observed because of this trend, offering consumers a broad range of options.

## 6 CONCLUSION

To summarize, non‐alcoholic wine presents noteworthy opportunities within the beverage sector by appealing to health‐conscious individuals, broadening the consumer demographic, facilitating social gatherings, elevating dining experiences, promoting ingenuity, and supporting sustainability efforts. By optimizing the dealcoholizing techniques employed in winemaking and implementing precise control on dealcoholizing factors, such as temperature and pressure, it is possible to minimize the loss of volatile compounds. This optimization can assist winemakers in enhancing the sensory characteristics of dealcoholized wine, thereby making it more appealing and acceptable in the market. The crucial aspect is to achieve a harmonious equilibrium between decreasing the alcohol content and maintaining the desirable flavors, aromas, and overall quality of the wine. The evolving consumer preferences are anticipated to fuel growth and innovation in the dealcoholized wine market, owing to the increasing demand for high‐quality, flavorful, and low‐alcohol or alcohol‐free alternatives.

## AUTHOR CONTRIBUTIONS

**Wasim Akhtar**: Writing–original draft; investigation; visualization; conceptualization. **Adriana Teresa Ceci**: Writing–review and editing; investigation; conceptualization. **Edoardo Longo**: Conceptualization; writing–review and editing; supervision. **Marco Adolfo Marconi**: Conceptualization; writing–review and editing. **Francesco Lonardi**: Conceptualization; writing–review and editing. **Emanuele Boselli**: Conceptualization; writing–review and editing; funding acquisition; project administration; resources; supervision.

## CONFLICT OF INTEREST STATEMENT

The authors declare no conflicts of interest.
